# Differential Responses of Cecal Microbiota to Fishmeal, *Eimeria* and *Clostridium perfringens* in a Necrotic Enteritis Challenge Model in Chickens

**DOI:** 10.1371/journal.pone.0104739

**Published:** 2014-08-28

**Authors:** Dragana Stanley, Shu-Biao Wu, Nicholas Rodgers, Robert A. Swick, Robert J. Moore

**Affiliations:** 1 Central Queensland University, School of Medical and Applied Sciences, Rockhampton, Queensland, Australia; 2 Australian Animal Health Laboratory, CSIRO Biosecurity Flagship, Geelong, Victoria, Australia; 3 Poultry Cooperative Research Centre, University of New England, Armidale, New South Wales, Australia; 4 School of Environmental and Rural Science, University of New England, Armidale, New South Wales, Australia; University of Arizona, United States of America

## Abstract

*Clostridium perfringens* causes enteric diseases in animals and humans. In poultry, avian-specific *C. perfringens* strains cause necrotic enteritis, an economically significant poultry disease that costs the global industry over $2 billion annually in losses and control measures. With removal of antibiotic growth promoters in some countries this disease appears to be on the rise. In experimental conditions used to study disease pathogenesis and potential control measures, reproduction of the disease relies on the use of predisposing factors such as *Eimeria* infection and the use of high protein diets, indicating complex mechanisms involved in the onset of necrotic enteritis. The mechanisms by which the predisposing factors contribute to disease progression are not well understood but it has been suggested that they may cause perturbations in the microbiota within the gastrointestinal tract. We inspected changes in cecal microbiota and short chain fatty acids (SCFA) induced by *Eimeria* and fishmeal, in birds challenged or not challenged with *C. perfringens*. *C. perfringens* challenge in the absence of predisposing factors did not cause significant changes in either the alpha or beta diversity of the microbiota nor in concentrations of SCFA. Moreover, there was no *C. perfringens* detected in the cecal microbiota 2 days post-challenge without the presence of predisposing factors. In contrast, both fishmeal and *Eimeria* caused significant changes in microbiota, seen in both alpha and beta diversity and also enabled *C. perfringens* to establish itself post challenge. *Eimeria* had its strongest influence on intestinal microbiota and SCFA when combined with fishmeal. Out of 6 SCFAs measured, including butyric acid, none were significantly influenced by *C. perfringens*, but their levels were strongly modified following the use of both predisposing factors. There was little overlap in the changes caused following *Eimeria* and fishmeal treatments, possibly indicating multiple routes for progressing towards clinical symptoms of necrotic enteritis.

## Introduction

It has been more than half a century since necrotic enteritis (NE) was first reported in chickens by Parish *et al.* [1]. Despite decades of research, NE remains one of the major challenges in the poultry industry and is associated with extensive production losses worldwide [2]. Although *Clostridium perfringens* is clearly the pathogen responsible for NE, both field experience and efforts to experimentally reproduce the disease have shown that onset of NE is a complex process requiring one or a number of predisposing factors rather than just the presence of pathogenic *C. perfringens* strains [3], [4].

In experimental disease challenge trials it is necessary to introduce predisposing factors, such as *Eimeria* co-infection, high protein feed (including fishmeal), indigestible non-starch polysaccharides, controlled immunosuppression or deliberate stressing of birds, to produce clinical symptoms in a substantial proportion of challenged birds [4]. Moreover, healthy birds often carry strains of *C. perfringens* without showing any clinical symptoms of NE. It is now clear that most of the *C. perfringens* strains colonizing healthy birds are non-pathogenic strains incapable of inducing NE. It has also been suggested that other bacteria may play a role in disease onset: for instance a significant reduction in *Weissella confusa* has been noted in *C. perfringens* challenged birds while uncultured mollicutes associated with human intestinal problems increased by 3.7 fold in NE birds [5]. Reduction of *Lactobacillus johnsonii* by NE [5] and independently by fishmeal [6] has also been reported.

The ban on the use of antibiotics as growth promoters in Europe has highlighted the growing need for alternatives to antibiotic supplementation as a way of controlling NE in poultry. It has been reported that addition of some short chain fatty acids (SCFAs) to chicken diets has the potential to help birds resist NE [7], [8]. It has also been noted that butyrate producing bacterial phylotypes are reduced in the intestinal microbiota during the onset of NE [5]. Interestingly SCFAs, especially butyrate, are also reduced in human colitis patients [9], [10]. Butyrate aids mucosal immunity and differentiation of colonic regulatory T cells [11] and it has the ability to attenuate symptoms of colitis [12].

It has been recently shown that *Eimeria* co-infection and high fishmeal diets induce very significant changes in cecal microflora [6]. The present study was undertaken to further investigate the impact of fishmeal and *Eimera* on cecal microflora and SCFA changes in birds challenged and unchallenged with pathogenic *C. perfringens*. A 2 × 2 × 2 factorial arrangement of treatments was employed with fishmeal (− or +), *Eimeria* (− or +) and *C. perfringens (− or +)* as factors. Cecal microbial abundance in all treatment groups was correlated with concentrations of short chain fatty acids. The results produced in the present study suggest that, by itself, the pathogenic *C. perfringens* strain was unable to cause any significant changes in the cecal microbiota, and furthermore it demonstrated a total inability to establish itself and remain in the microbial community 2 days post-challenge, without the help of predisposing factors. We also found butyrate levels as well as other SCFAs were not significantly influenced by *C. perfringens* but were strongly influenced by the predisposing factors. This study identified microbiota and SCFA changes that correlated with the use of predisposing factors, which may be important in the successful induction of NE in the experimental systems. A deeper understanding of the factors affecting disease progression in experimental systems is likely to assist in the development of alternative control strategies for implementation in the field.

## Materials and Methods

### Animal trial

**Figure 1:**
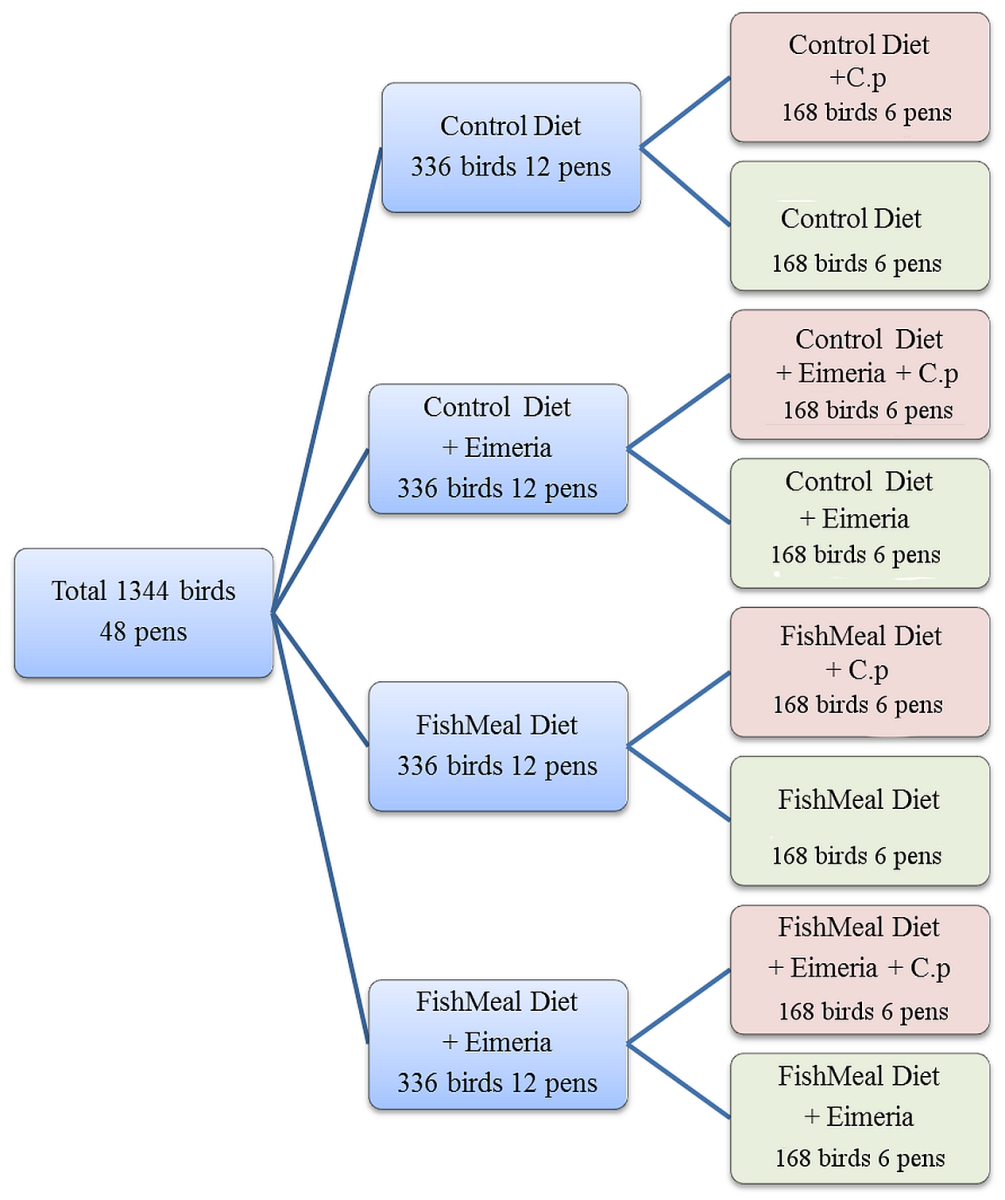
Diagram of the experimental design used in the animal trial. “C.p” was used to abbreviate *C. perfringens*.

The animal trial was performed using day-old Cobb 500 male broiler chickens obtained from Baiada Country Road Hatchery, Tamworth, NSW, Australia. Chickens were vaccinated and initially handled as previously described [6]. Briefly, birds were raised in floor pens in a temperature controlled room. Water and food were provided ad libitum. The treatment groups were as follows: control (CTRL), high (25% w/w) fishmeal (Skretting, Tasmania) feed (FM), *Eimeria* (E) challenge group and a group with both fishmeal and *Eimeria* (E+FM). For *Eimeria* challenge chicks were gavaged *per os*, with a suspension of 5,000 oocysts of *E. acervulina* and *E. maxima*, and 2,500 oocysts of *E. brunetti* (Bioproperties Pty., Glenorie, NSW, Australia) in 1 mL PBS with unchallenged birds just receiving PBS. Each of the four main groups were divided into 6 pens of unchallenged and 6 pens of birds challenged with *C. perfringens* (strain EHE-NE18). At 14 and 15 days birds to be challenged were inoculated *per os* with 1 mL of *C. perfringens* suspension at a concentration of 10^8^–10^9^ CFU/mL. Birds in unchallenged groups received 1 mL of sterile thioglycollate broth (Oxoid). Two birds per pen were euthanized and the cecal digesta sampled on 16 d for subsequent microbiota analysis. Collected cecal digesta from two birds pooled to represent each pen was snap-frozen in liquid nitrogen and then stored at −20°C until required for DNA extraction. The NE lesion score was determined according to Prescott, *et al.* [13]. The fact that each predisposing factor had unchallenged control and challenged group of birds allowed us to compare, for the first time, the success of challenge to concomitant changes in microbiota and SCFAs induced in each of treatment groups. The graphical presentation of the design is given in Figure 1.

### Animal ethics statement

All the birds were raised and handled humanely, and the animal trial was approved by the Animal Ethics Committee of the University of New England (AEC10/129). All procedures were according to “Australian code of practice for the care and use of animals for scientific purposes” (Australian Agricultural Council, 1997) and the “Australian model code of practice for the welfare of animals, Domestic Poultry” (Standing Committee on Agriculture and Resource Management, 1995).

### Analysis of short chain fatty acids and enumeration of bacteria by culturing

The analysis of SCFAs and enumeration of bacteria followed the methods used by Wu *et al.* [14]. Briefly, SCFAs and lactate concentrations were measured in a 2.0 g cecal sample by gas chromatography using 2-ethylbutyric acid as an internal standard. The concentrations of organic acids were determined using a Varian CP3800 gas chromatograph (Varian Analytical Instruments, Palo Alto, CA). Varian Star 5.52 chromatography workstation (integration system) software (Varian Analytical Instruments) was used to process all the data. Total SCFA concentration was calculated by the sum of all the short chain organic acids including lactate measured in a sample, expressed as µmol/g digesta, and individual organic concentration was expressed as the level relative to the total SCFA (%). For the enumeration of the bacteria, approximately 1 g of cecal digesta sample was collected in reduced salt solution, and then homogenized for 2 min in CO_2_-flushed plastic bags using a MiniMixH bag mixer (Interscience, St. Nora, France). The homogenized sample was serially diluted in six 10-fold steps. Aliquots (100 µL) from appropriate dilutions were plated on the following media to culture the respective bacteria; total anaerobic bacteria on Wilkins-Chalgren anaerobic agar (Oxoid, UK); coliform bacteria and lactose-negative enterobacteria on MacConkey agar (Oxoid, UK); lactic acid bacteria on de Man, Rogosa, and Sharpe agar (Oxoid, UK); and lactobacilli on Rogosa agar (Oxoid, UK). Bacterial numbers were expressed as log_10_ CFU/g digesta. Significant changes in SCFA profiles and cultured bacteria CFU were identified using ANOVA followed by Tukeys HSE test in R software.

### Microbiota analysis

Cecal DNA was extracted as previously described [15] with QIAamp DNA stool kit (Qiagen). The DNA amplification procedure was carried out as previously described [16]. The primers used (forward primer [17], 5′ AGAGTTTGATCCTGG 3′; reverse primer a truncated version of W31 [18], 5′ TTACCGCGGCTGCT 3′) were selected to amplify the V1 to V3 region of the 16S rRNA gene. Sequencing was performed on a Roche/454 FLX+ Genome Sequencer according to manufacturer’s instructions. The data pre-processing was done using PyroBayes [19] followed by further analysis in Qiime v1.6.0 [20] using usearch, uchime and uclust algorithms [21] at sequence similarity threshold of 97% and Blast taxonomy assignments. Sequences were trimmed using the parameters: minimum average quality score of 25, sequence lengths of 300–600 bases, no ambiguous sequences, and a maximum run of 6 bases in homopolymers. Low abundance operational taxonomic units (OTUs) were removed if they had less than 5 sequences and presence in less than 3 samples. The OTU abundance table was normalised using the average values from 100 rarefactions. Phylogenetic analysis was carried out using a variety of tools including the EzTaxon database [22], GreenGenes database (http://greengenes.lbl.gov/), BlastN 2.2.20 [23] and an R phylogenetic package, Ade4 (Analysis of Data functions for Ecological and Environmental data in the framework of Euclidean Exploratory) [24].

## Results

### Necrotic enteritis induction

**Figure 2:**
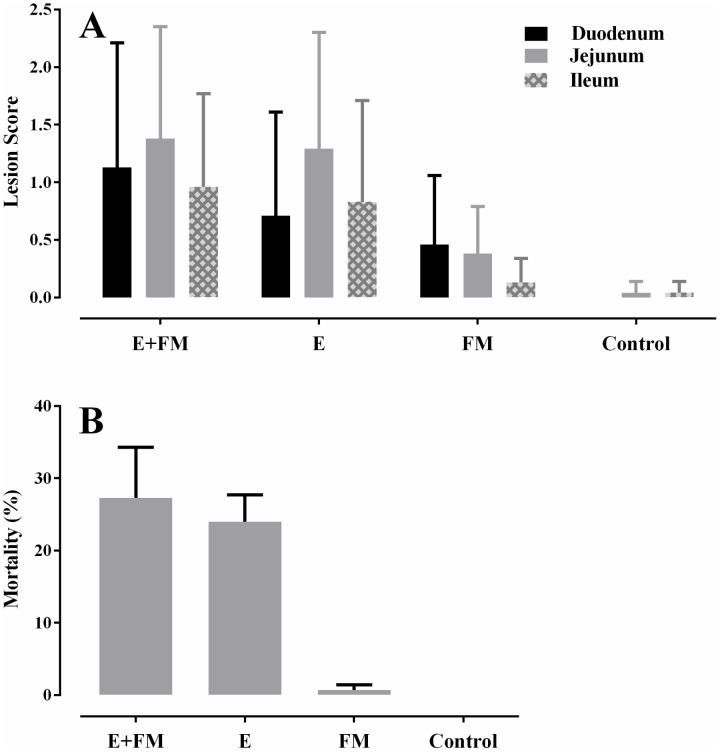
Barcharts showing lesion scores (A) and mortality (B) in groups challenged with *C. perfringens*. The groups not challenged with *C.perfringens* had no notable lesions or mortality. Lesions were scored as described in [13]. The bars show the standard errors. Missing bars indicate no lesions or mortality.

Necrotic enteritis lesion scores in the small intestine of birds and NE induced mortalities observed in the different treatment groups are shown in Figure 2. In all sections of the GIT inspected, the highest NE lesion scores and mortality were recorded in the group challenged in the presence of both fishmeal and *Eimeria* (E+FM+Cp). The *Eimeria* challenge group also had high lesion scores. Lesions were detected in the challenged birds fed fishmeal, however, the NE induced mortality rate for this group was very low. Both lesion score and mortality data showed that challenge with *C. perfringens* without the presence of predisposing factors was not capable of producing clinical symptoms of NE. Other aspects of this trial, in particular the effects of different treatments on bird productivity before *C. perfringens* challenge, have been reported in a separate publication [6]. The NE lesion scores have been important to consider both in the current work with regard to microbiota changes and in the other publication in the context of productivity and hence different presentations of the lesion and mortality data are given.

### Impact of treatments on microbiota diversity

The experimental design (Figure 1) allowed us to investigate the interaction of predisposing factors and *C. perfringens* infection on the overall microbiota composition within the ceca of treated birds. To inspect the effect of *Eimeria*, fishmeal and *C. perfringens,* individually and in combinations, on diversity and structure of cecal microbial communities, we have inspected alpha diversity measures (sample OTU richness) including chao1, dominance, Shannon, equitability, observed species and phylogenetic diversity using Qiime. Across all samples and groups the inspected alpha metrics were not significantly (P<0.05) influenced by the presence vs. absence of *C. perfringens* challenge. The influence of presence vs. absence of fishmeal was detected only in phylogenetic diversity metrics (P = 0.006) while other metrics fell short of the threshold P-value for statistical significance. The greatest influence on alpha diversity was observed with the presence vs. absence of *Eimeria* with the reduction in diversity in the *Eimeria* groups giving P-values ranging from 0.001 for phylogenetic diversity to 0.008 for the equitability metric (Tables S1 and S2 in File S1). We have further dissected this finding to demonstrate that this difference is caused by a sample group containing both *Eimeria* and *C. perfringens* rather than other groups of samples containing *Eimeria* (E, E+FM or E+FM+Cp) (Figure S1 in File S1). The highest difference in alpha diversity between any two treatment groups was between control (no *Eimeria*, no fishmeal, no *C. perfringens*) vs. E+C groups. This comparison significantly affected all inspected alpha diversity measures with P-values ranging from 0.036 to 0.006. A bar-chart of species abundance (Figure S2 in File S1) demonstrates that the effect of combined *Eimeria* and *C. perfringens* on cecal microbiota composition was very pronounced.

**Figure 3:**
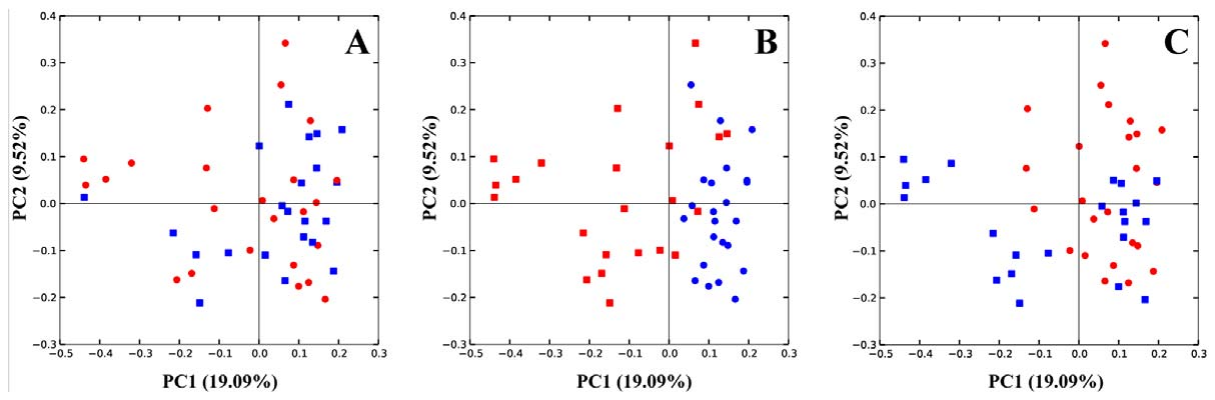
UnweightedUniFracPCA plot. Panel A is coloured by presence (red) and absence (blue) of *C. perfringens* in samples; in panel B samples are coloured according to presence (red) or absence (blue) of *Eimeria* and in panel C the same samples are coloured by presence (red) or absence (blue) of fishmeal diet.

**Figure 4:**
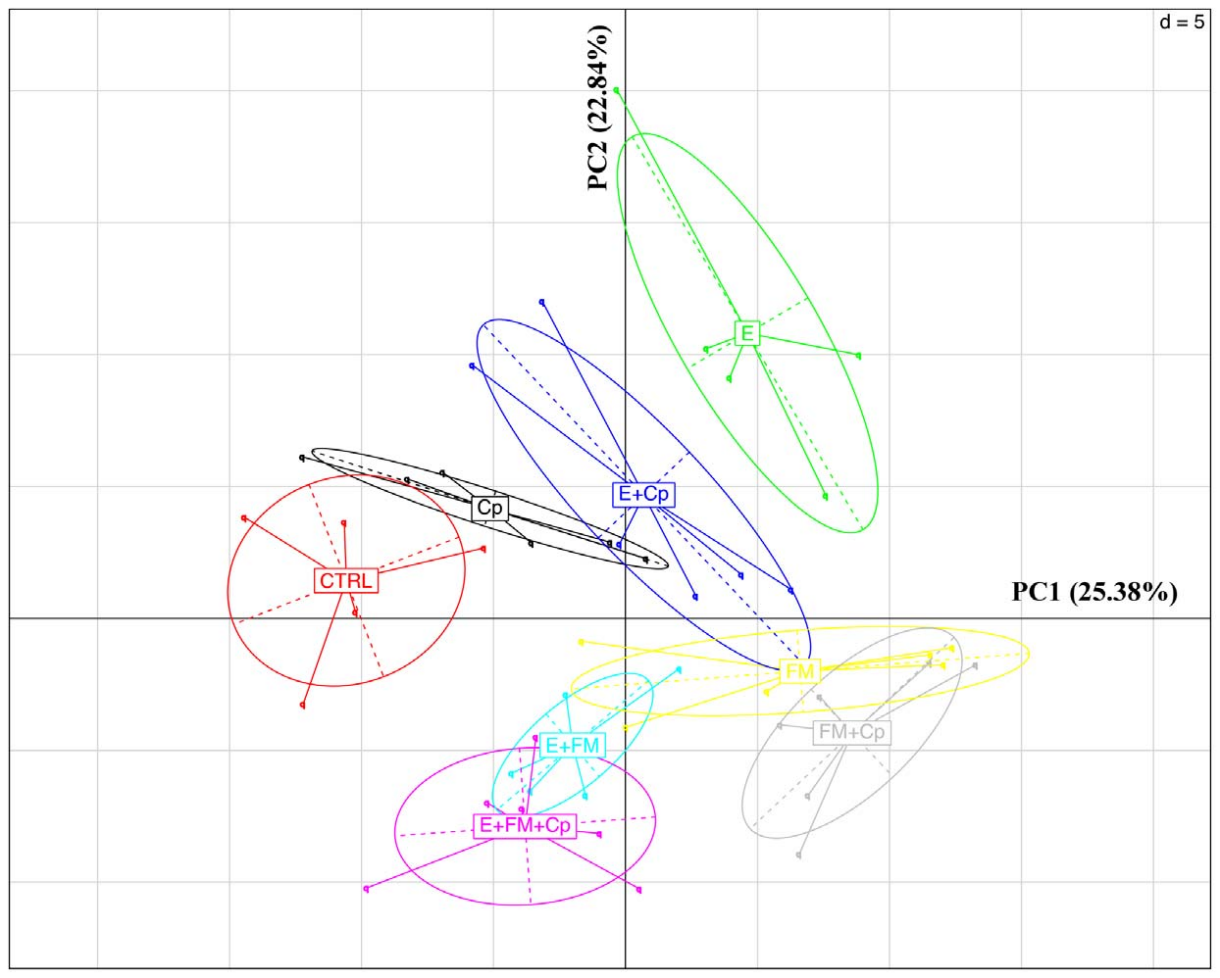
PCA plot generated using the Ade4 R phylogenetic package [24]. Principal Component Analysis combined with duality diagram functions (dudi) [32] was used to perform Between-Class Analysis (bca) with respect to sample assignment to treatment groups. Monte Carlo test based on 999 permutations demonstrates that groups are significantly (Monte Carlo P-value = 0.003) different. The graph shows sample and group relationship with respect to microbiota structure. The OTU table used for the analysis was 100 times rarefied (normalised) and OTUs with abundance lower than 0.001 were removed.

Observing beta diversity (between sample OTU difference) using weighted and unweighted UniFrac and ADONIS statistics at 999 permutations, more differences between groups were observed in presence and absence (unweighted UniFrac) than in abundance (weighted UniFrac) of OTUs. Unweighted UniFrac demonstrated significant differences in OTUs within the cecal microbiota correlating with the presence vs. absence of *Eimeria* (p<0.001) and also by the presence vs. absence of fishmeal (P<0.001), while *C. perfringens* presence/absence produced insignificant (P = 0.058) changes to the microbiota (Figure 3). Based on weighted UniFrac, *Eimeria* had the strongest influence (P<0.001) followed by fishmeal (P = 0.036) with again insignificant influence of presence of *C. perfringens* (P = 0.168). Finally, multivariate phylogenetic analysis (Ade4-generated PCA plot) visualised the differences between the groups using between class analyses, confirming the above results (Figure 4). The pyrosequencing data files used for this study have been deposited in the MGRAST metagenomics database (http://metagenomics.anl.gov/) with accession numbers 4567980.3 to 4568025.3.

### Phylogeny of bacteria altered by predisposing factors and NE challenge

**Figure 5:**
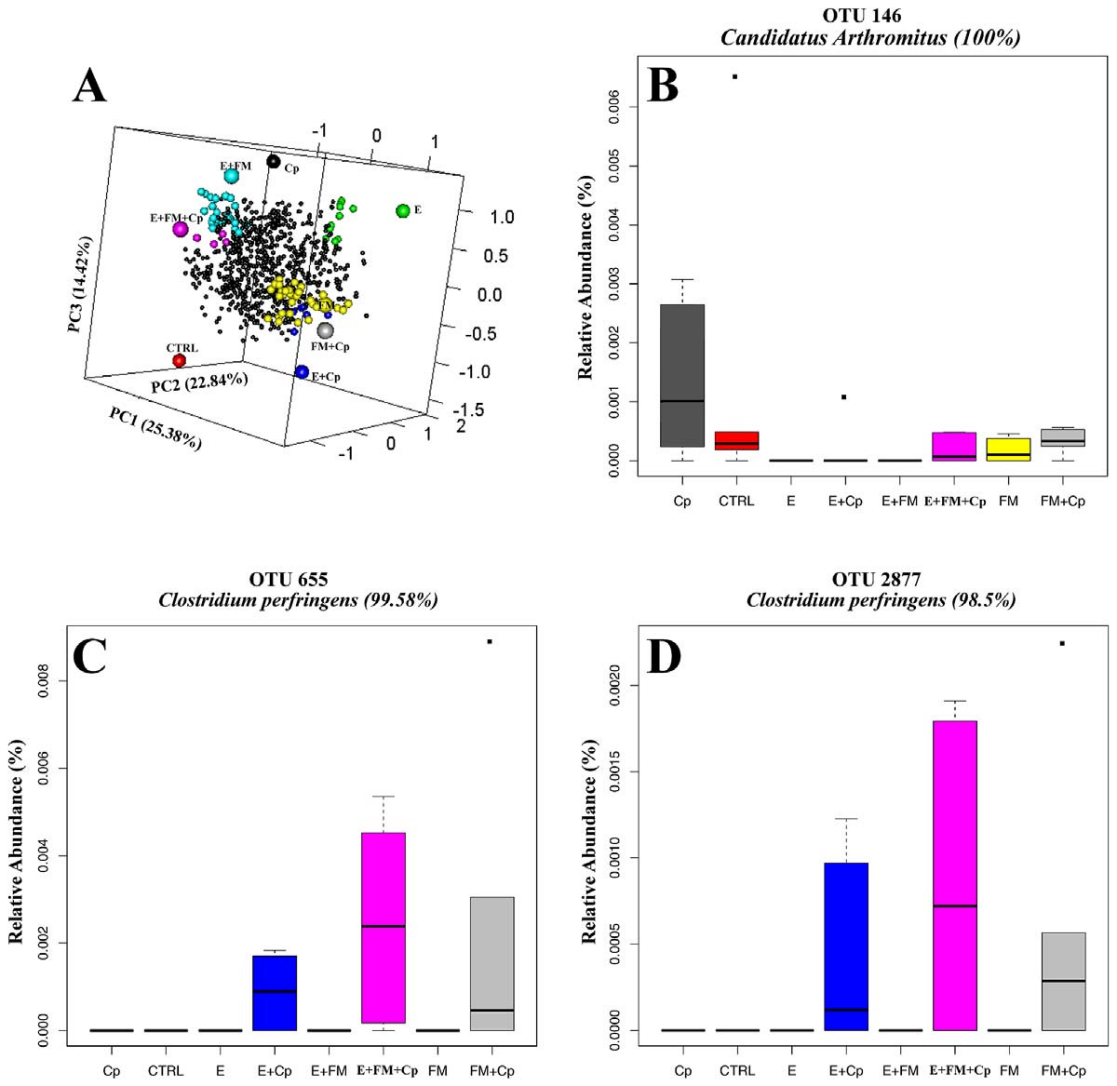
Duality diagram function and between class analyses presented in the form of a 3D PCA plot and selected significant OTUs in form of boxplots. The 3D graph (A) shows the relationship between sample groups and OTUs driving the sample differences. OTUs significantly different between groups were selected in Ade4 using the relative abundance table and confidence level of 95%; only OTUs with total abundance higher than 0.001 were retained in the analysis. The large circles represent treatment groups while small black circles represent OTUs. Only OTUs that are significantly associated with differences between groups are enlarged and coloured in the same colour as the sample they are distinguishing from others. Some of the significant OTUs are given in views B, C and D while all OTUs selected by Ade4 analysis are given in Figure S3. The boxes represent the limits of the second and third quartiles; the whiskers indicate the data within 1.5 times the interquartile range and the dots are outliers.

We used Ade4 between group multivariate phylogenetic analysis to identify the differences between treatment groups and to identify OTUs that most strongly influence between group differences at a confidence level cut-off of 95%. Figure 5 is a graphical representation of this analysis in the form of a 3D PCA plot (Figure 5A) and boxplots showing 3 selected OTUs across the treatment groups (Figure 5B–D). The 3D PCA plot presented in Figure 5A shows the relative relationship of treatment groups to OTUs. Based on the results of Ade4 analysis, the most affected phylotypes were related to the major chicken intestinal genera *Clostridium*, *Lactobacillus*, *Eubacterium* and *Ruminococcus*. OTU146 has 100% sequence similarity with the type strain of *Candidatus* Arthromitus, later renamed *Candidatus* Savagella [25], and was present at much higher frequency within the microbiota of *C. perfringens* challenged birds on the control diet than in all other groups (Figure 5B). Figure 5B also shows the presence of this OTU in three fishmeal containing groups and absence in three groups containing *Eimeria*. Among differentially abundant OTUs were OTU2877 and OTU655 with 98.5% and 99.58% similarity respectively to *C. perfringens* type strain (EzTaxon) (Figure 5C and 5D). *C. perfringens* was present in the challenged groups with predisposing factors, either used separately or together, but surprisingly was completely absent in all challenged birds that were not treated with predisposing factors (Figure 5C–D). All other OTUs marked as significantly different between groups in Ade4 analysis are provided in the form of bar-chart in a supplementary figure (Figure S3 in File S1) and their representative sequences are publically available on EMBL database under accession numbers HG810792 to HG810850.

### SCFA production is altered during the challenge

**Figure 6:**
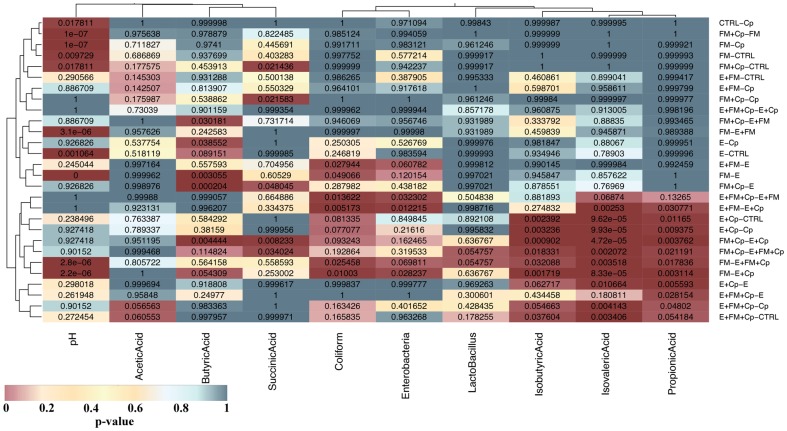
Table of TukeyHSD corrected p-values given in form of a heatmap. ANOVA was calculated for differences between groups for each of the SCFAs, pH and cultured bacterial counts (columns of the heatmap). ANOVA was followed by TukeyHSD honesty test to calculate p-values (shown in each cell of the heatmap) for each to each group-to-group comparison (rows). The heatmap was coloured by p-values according to the key below the graph in order to emphasise significant differences (in dark red). For example the lower right corner cell shows that groups E+FM+Cp and CTRL group (labelled E+FM+Cp – CTRL on the graph) are showing slight differences (TukeyHSD corrected P-value = 0.054184) in concentration of propionic acid. The clustering of heatmap rows and columns was done to identify similarities in SCFA changes between conditions and was based on Euclidean distance and complete agglomeration method in R package ggplot. This identified a hot-spot in the lower right corner of the heatmap of sample comparisons showing significant differences in isobutyric, isovaleric and propionic acid.

Significant changes in SCFA profiles were produced by combinations of *C. perfringens* challenge and predisposing factors (Figure 6). The challenge with *C. perfringens* caused significant changes only in pH (P = 0.0178) with no significant changes in any of the SCFA inspected (P-values ranging from 0.97 to 1). Figure 6 shows that the majority of significant differences were observed in concentrations of isobutyric, isovaleric and propionic acid (lower right corner heat spot in Figure 6); in all of these comparisons at least one group of the 2 groups compared was challenged with *C. perfringens* and at least one group of the pair with *Eimeria*. There are no comparisons in this “hot spot” that did not contain at least one predisposing factor. Bar-charts showing concentration differences in all SCFAs measured are provided in Figure S4.

**Figure 7:**
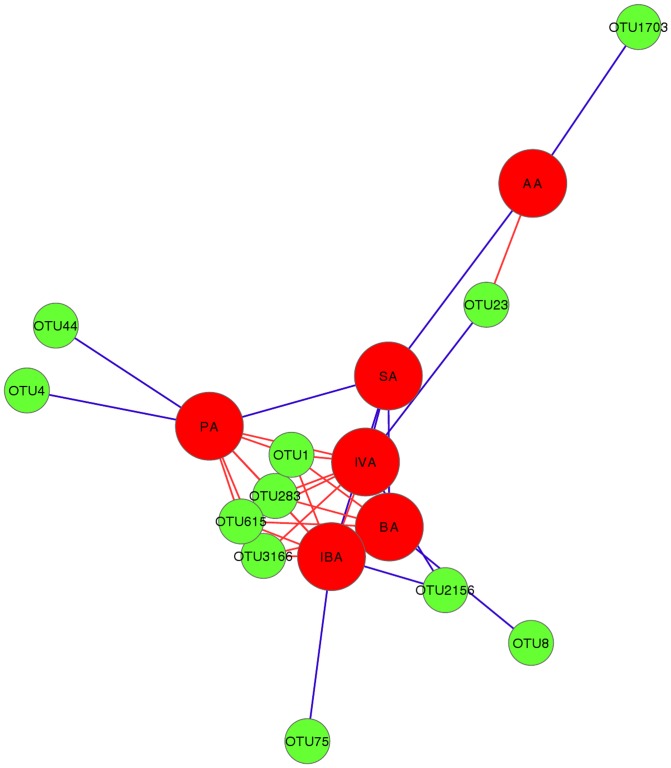
Spring embedded network showing Pearson correlations between SCFA and OTUs (analysed in Qiime). Edge length in the network is proportional to Pearson correlation r value; blue edge is used for negative and red edge for positive correlation. SCFA are represented with big red circles and shortened as AA = acetic Acid, SA = Succinic Acid, IVA = isovaleric Acid, BA = butyric Acid, IVA = IsoValeric Acid and PA = propionic Acid. OTUs are represented with green circles.

Correlation based on Pearson distance between OTU abundances and concentrations of SCFAs (Qiime) is presented as a Cytoscape-visualised Spring Embedded network in Figure 7. Only significant correlations with P-values<0.05 were included in the graph. The graph displays a group of OTUs, OTU1, OTU283, OTU615 and OTU3166, all identified as *L. johnsonii* with sequence similarities of 100, 99, 98 and 94% respectively, which are strongly anti-correlated with propionic, butyric, isovaleric and isobutyric acid concentrations. All 4 of these SCFAs were increased in the presence of *Eimeria* and *C. perfringens* together, with either the presence or absence of fishmeal. OTU1713, identified as *L. reuteri* (99% identity), was positively correlated with acetic acid concentrations. An unknown *Clostridium,* closest to *Clostridium methylpentosum* (89% identity), was positively correlated with butyric acid.

The numbers of culturable Coliforms and Enterobacteria were increased in the presence of *Eimeria* while counts of total *Lactobacillus* were not significantly changed (Figure S4 in File S1, Figure 6). pH varied significantly between the groups appearing to be reduced in *Eimeria* containing groups and increased in fishmeal fed groups (Figure S4 in File S1, Figure 6).

## Discussion

At present, fishmeal and *Eimeria*, alone or combined, are the most widely used predisposing factors in NE challenge models. The mechanisms of *C. perfringens* interactions with *Eimeria* and fishmeal diet remain poorly understood but one hypothesis is that these factors disrupt the gastrointestinal environment creating disturbances in the resident microbiota and thus allowing the proliferation of *C. perfringens* by either providing nutrients or favourable ecological niches [4]. The data presented here demonstrate, for the first time, that a *C. perfringens* challenge alone is not capable of causing any significant perturbations of intestinal microbiota in birds. However, the predisposing factors *Eimeria* and/or fishmeal are able to have a marked effect on the microbiota composition in the caeca of birds. The strongest influence on alpha and beta diversity of microbiota was recorded when *Eimeria* was used in combination with *C. perfringens*. This is in agreement with previously published studies that investigated mortality of chickens under NE challenge induced with either *Eimeria* or fishmeal [14]. Wu *et al*., [14] noted the strongest influence on NE induced mortalities when *Eimeria* and fishmeal were combined, while fishmeal alone did not significantly increase mortality of chickens challenged with *C. perfringens*. Although fishmeal had less significant effects on alpha and beta diversity than *Eimeria,* it still caused significant microbiota changes and supported establishment of *C. perfringens* in the cecal community and was thus capable of providing an environment better suited to *C. perfringens* infection.

One of the most surprising findings was that administration of *C. perfringens* on its own did not cause the pathogen to maintain and establish itself. *C. perfringens* was not identified in the 16S sequencing data in any of the birds in the unchallenged control group or the *C. perfringens* challenged group without predisposing factors, while all groups containing predisposing factors, E, FM or E+FM, showed significant presence of *C. perfringens* in all birds post-challenge (Figure 5C and D).

Administration of *C. perfringens* was correlated with an increase in the abundance of OTU146, identified by EzTaxon as 100% identical to *Candidatus* Arthromitus (accession number X80834), recently renamed to *Candidatus* Savagella based on 16S phylogenetic analysis. This group of segmented bacteria are found in gut microbiota of vertebrates like chickens, rodents and fish and are anchored to the gut epithelial cells in the ileum and play an important role in modulating host immune responses [25]. They increase, activate, and coordinate maturation of epithelial lymphocytes [26]–[28], and induce IgA secreting cells [29]. It was surprising to see this OTU increased only in the *C. perfringens* infected group when predisposing factors were not used but its abundance was not altered in the presence of *C. perfringens* when predisposing factors were also applied. This OTU was detected in only one bird from the three treatment groups containing *Eimeria*; in all other birds in these groups it was absent from the 16S analysis. If *Eimeria* is capable of removing or reducing this immune modulating bacterium to a level lower than the detection limit, its removal could start a chain reaction to unbalance gut mucosal immunity. This could represent a novel way in which *Eimeria* modulates host immunity to increase infectivity. A reduction in *Candidatus* Savagella was not observed in the *Eimeria* group that also had both fishmeal and *C. perfringens*. The group only treated with *C. perfringens* challenge, that strongly induced OTU146, did not develop any clinical symptoms of NE and was capable of suppressing *C. perfringens* establishment in the gut. Whether the increase in *Candidatus* Savagella had any protective role, or was just a coincidental bystander to the challenge needs to be further investigated. Interactions of *Eimeria* and this phylotype are also of potential interest.

The previously reported perturbations in the *Lactobacillus* genus due to *C. perfringens* [5] or predisposing factors [6] were also noted in the present study. *L. acidophilus* (OTU 10, 100% identity, OTU1669, 99.14% identity) and *L. johnsonii* (OTU 823, 98.76% identity) which were previously reported as reduced by NE in a fishmeal challenge model [5], [6] were again reduced in the present study when birds were challenged with *C. perfringens* in the presence of fishmeal. However, both of these *Lactobacillus* phylotypes flourished in *C. perfringens* challenged birds in the presence of *Eimeria*. The few other OTUs confidently (>97%) identified as *L. johnsonii* were not reduced by fishmeal but were all consistently induced by *Eimeria* and *C. perfringens*. This, together with many other phylotype examples (Figure S3), indicate core differences in the influence of *C. perfringens* challenge following the application of *Eimeria* and fishmeal predisposing factors. It is well documented that *Lactobacillus* members have very strict and fastidious requirements for metabolites in their environment, including SCFAs [30]. Groups containing *Eimeria* and/or fishmeal all showed reduced concentrations of acetic acid while isobutyric, butyric, and isovaleric acid concentrations were significantly increased in all groups containing *Eimeria*. Since the presence of *Eimeria* influenced intestinal SCFA concentrations the most, it is likely that SCFA fluctuations influenced some phylotypes including *L. acidophilus* and *L. johnsonii* in the opposite manner to other groups. SCFA vs OTU correlation network analysis indicated that abundances of OTUs corresponding to *L. johnsonii* and *L. reuterii* were strongly correlated with SCFA concentrations, which could also point to another mechanism contributing to *Eimeria*’s influence on NE, as perturbations within genus *Lactobacillus* were previously reported as a response to NE challenge with [6] and without [5] presence of *Eimeria*. This indicates that more than one route can lead to successful *C. perfringens* infection of birds followed by clinical symptoms. Although some of the changes induced in gut microbiota during *C. perfringens* infection have been reproducibly observed across trials it is likely that other aspects of the microbiota perturbations will vary from trial to trial given the very significant differences in the gut microbiota that has been observed in different trials and even within groups of birds [31].

Based on the present study, we can conclude that the pathogenic *C. perfringens* strain used in this study cannot, on its own, establish itself as a member of the chicken cecal microbiota but needs other factors (e.g. fishmeal or *Eimeria* infection) in order to successfully induce the disease. Whether the principal roles of these predisposing factors in the disease models are to change intestinal microbiota and thus intestinal health and metabolite profiles or these microbiota changes are a side effect remains to be further investigated. The data collected to date strongly correlate a compromised microbial community with the ability of *C. perfringens* to cause an infection. It is very likely that other predisposing factors may compromise intestinal health using different molecular strategies. Investigating *C. perfringens* challenge using other predisposing factors, such as targeted immune system compromising, could shed more light on mechanisms of necrotic enteritis onset as well as possible strategies for controlling this important disease.

## Supporting Information

File S1 Contains Table S1, Comparison of alpha diversity statistics between treatment groups. The default Qiime settings were used based on a nonparametric two-sample t-test (using 100 times rarefied OTU table and 1000 Monte Carlo permutations to calculate P-values). The upper right section of the matrix, shaded light grey, indicates the p-values for the dominance metric. The lower left section of the matrix, shaded darker grey, indicates the alpha diversity comparison using the observed species metric. P values of less than 0.05 are in bold. Table S2, Detection of statistically significant differences in alpha diversity induced by the different treatments. For each comparison multiple treatment groups were combined. For example, for the *Eimeria* treatment comparison all birds that were treated with *Eimeria*, regardless of fish meal or *C. perfringens* treatment, were compared with all birds that did not receive *Eimeria*. P values of less than 0.05 are in bold. Figure S1, Alpha rarefaction graphs of groups and treatments. Alpha rarefaction measures represented as Observed Species (A and C), Dominance (B and D), Doubles (E), and Equitability (F). The analysis on individual treatment group basis is shown in panels A and B. The analysis of combined data sets (e.g. all with *C. perfringens*, all with *Eimeria* or all with both) are shown in panels C–F and demonstrate the strong influence by the combination of *Eimeria* and *C. perfringens*. Figure S2, Barchart of OTU abundances at a species level. Figure S3, Boxplots of OTUs identified as differential by Ade4 analysis. Continued from main Figure 4. The boxes represent the limits of the second and third quartiles; the whiskers indicate the data within 1.5-fold of the interquartile range; the median is indicated by the horizontal line and the dots are outliers. Note: In order to fit the legend on the x axis the names of two groups are omitted for gray column (E+FM) and for brown column (FM) in boxplots. Figure S4, Boxplots showing SCFA, pH and cultured bacteria across the treatment groups. The blue column in the boxplots represents E+FM+Cp group. The boxes represent the limits of the second and third quartiles; the whiskers indicate the data within 1.5-fold of the interquartile range; the median is indicated by the horizontal line and the dots are outliers.
(DOCX)
