# Supplementary material for: Differential Responses of Cecal Microbiota to Fishmeal, Eimeria and Clostridium perfringens in a Necrotic Enteritis Challenge Model in Chickens
Source: PLoS One. 2014 Aug 28;9(8):e104739. doi: 10.1371/journal.pone.0104739 (PMC4148237; doi:10.1371/journal.pone.0104739)

**Interplay of *Clostridium perfringens* and the predisposing factors, dietary fishmeal and *Eimeria*, in the onset of necrotic enteritis – an intestinal microbiota perspective**

Dragana Stanley, Shu-Biao Wu, Nicholas Rodgers, Robert A. Swick, and Robert J. Moore

**Supplementary Data**

Table S1: Comparison of alpha diversity statistics between treatment groups 2

Table S2: Differences in alpha diversity induced by the different treatments 2

Figure S1: Alpha rarefaction measures are influenced by Eimeria 3

Figure S2: Barchart of OTU abundances at a species level 4

Figure S3: Boxplots of OTUs identified as differential by ade4 analysis 5

Figure S4: Boxplots showing SCFA, pH and cultured bacteria 14

**Table S1:** Comparison of alpha diversity statistics between treatment groups. The default Qiime settings were used based on a nonparametric two-sample t-test (using 100 times rarefied OTU table and 1000 Monte Carlo permutations to calculate P-values). The upper right section of the matrix, shaded light grey, indicates the p-values for the dominance metric. The lower left section of the matrix, shaded darker grey, indicates the alpha diversity comparison using the observed species metric. P values of less than 0.05 are in bold.

| Dominance | | | | | | | | |
| --- | --- | --- | --- | --- | --- | --- | --- | --- |
| Group | F | FECp | E | FCp | FE | ECp | Cp | Control |
| F | 1 | 0.28 | 0.7 | 1 | 0.56 | 0.168 | 1 | 1 |
| FECp | 0.308 | 1 | 1 | 1 | 1 | 1 | 0.56 | 1 |
| E | 0.056 | 1 | 1 | 1 | 1 | 1 | 1 | 1 |
| FCp | 1 | 1 | 1 | 1 | 1 | 1 | 1 | 1 |
| FE | 1 | 1 | 1 | 1 | 1 | 0.7 | 1 | 1 |
| ECp | 0.056 | 1 | 1 | 0.364 | 0.42 | 1 | 0.168 | 1 |
| Cp | 1 | 1 | 1 | 1 | 1 | **0.028** | 1 | 1 |
| Control | 1 | 1 | 1 | 1 | 1 | 0.42 | 1 | 1 |
| Observed Species | | | | | | | | |

**Table S2:** Detection of statistically significant differences in alpha diversity induced by the different treatments. For each comparison multiple treatment groups were combined. For example, for the *Eimeria* treatment comparison all birds that were treated with *Eimeria*, regardless of fish meal or *C. perfringens* treatment, were compared with all birds that did not receive *Eimeria*. P values of less than 0.05 are in bold.

| Comparison | Simpson | Dominance | Equitability | Observed species | Shannon | PD whole tree |
| --- | --- | --- | --- | --- | --- | --- |
| *Eimeria* (present vs absent) | **0.016** | **0.009** | **0.008** | **0.002** | **0.004** | **0.001** |
| *C. perfringens* (present vs absent) | 0.115 | 0.124 | 0.131 | 0.095 | 0.113 | 0.209 |
| Fish meal (present vs absent) | 0.085 | 0.089 | 0.074 | 0.054 | 0.064 | **0.006** |

**Figure S1:** Alpha rarefaction graphs of groups and treatments.

Alpha rarefaction measures represented as Observed Species (A and C), Dominance (B and D), Doubles (E), and Equitability (F). The analysis on individual treatment group basis is shown in panels A and B. The analysis of combined data sets (e.g. all with *C. perfringens*, all with *Eimeria* or all with both) are shown in panels C-F and demonstrate the strong influence by the combination of *Eimeria* and *C. perfringens*.


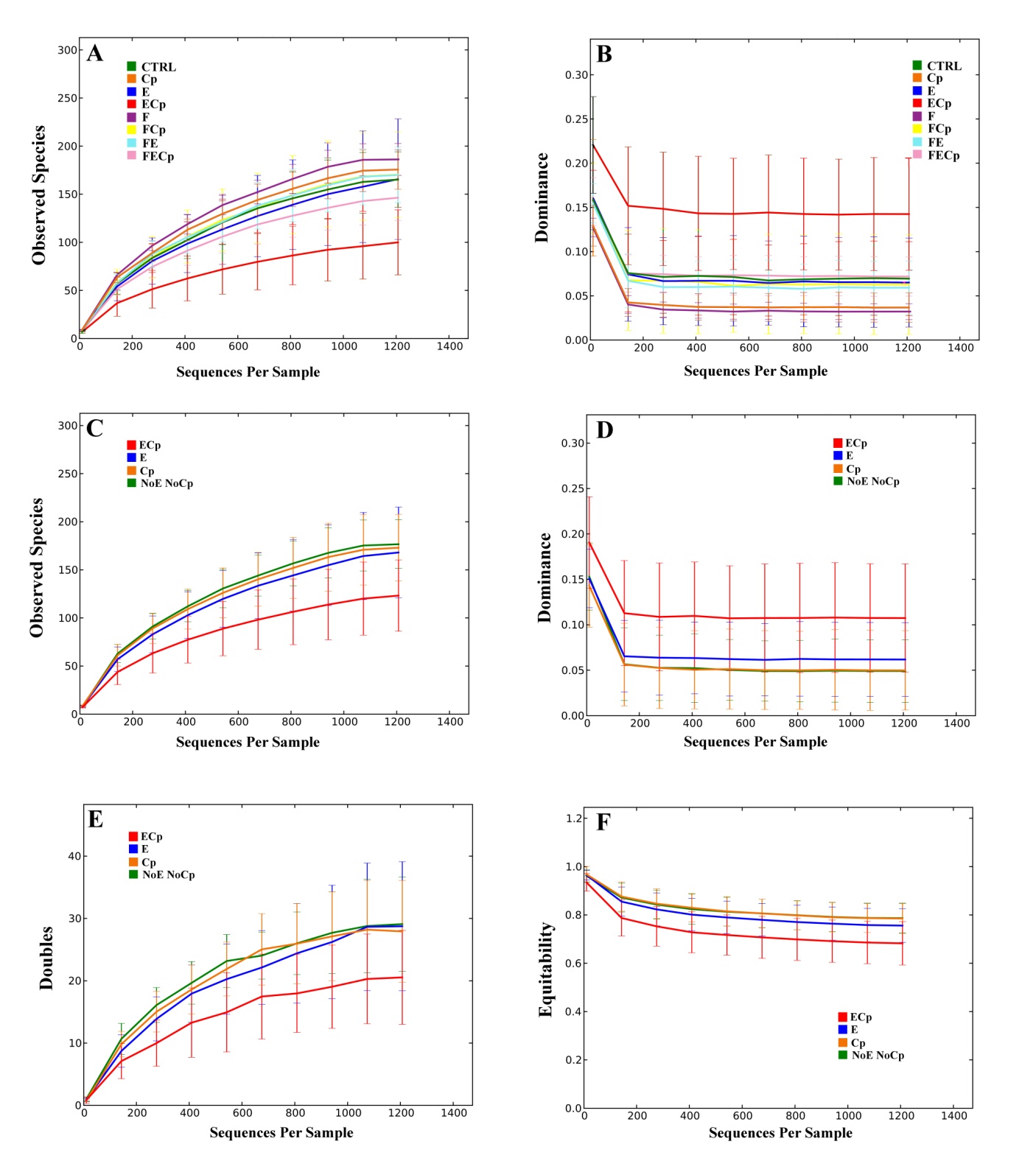


**Figure S2:** Barchart of OTU abundances at a species level


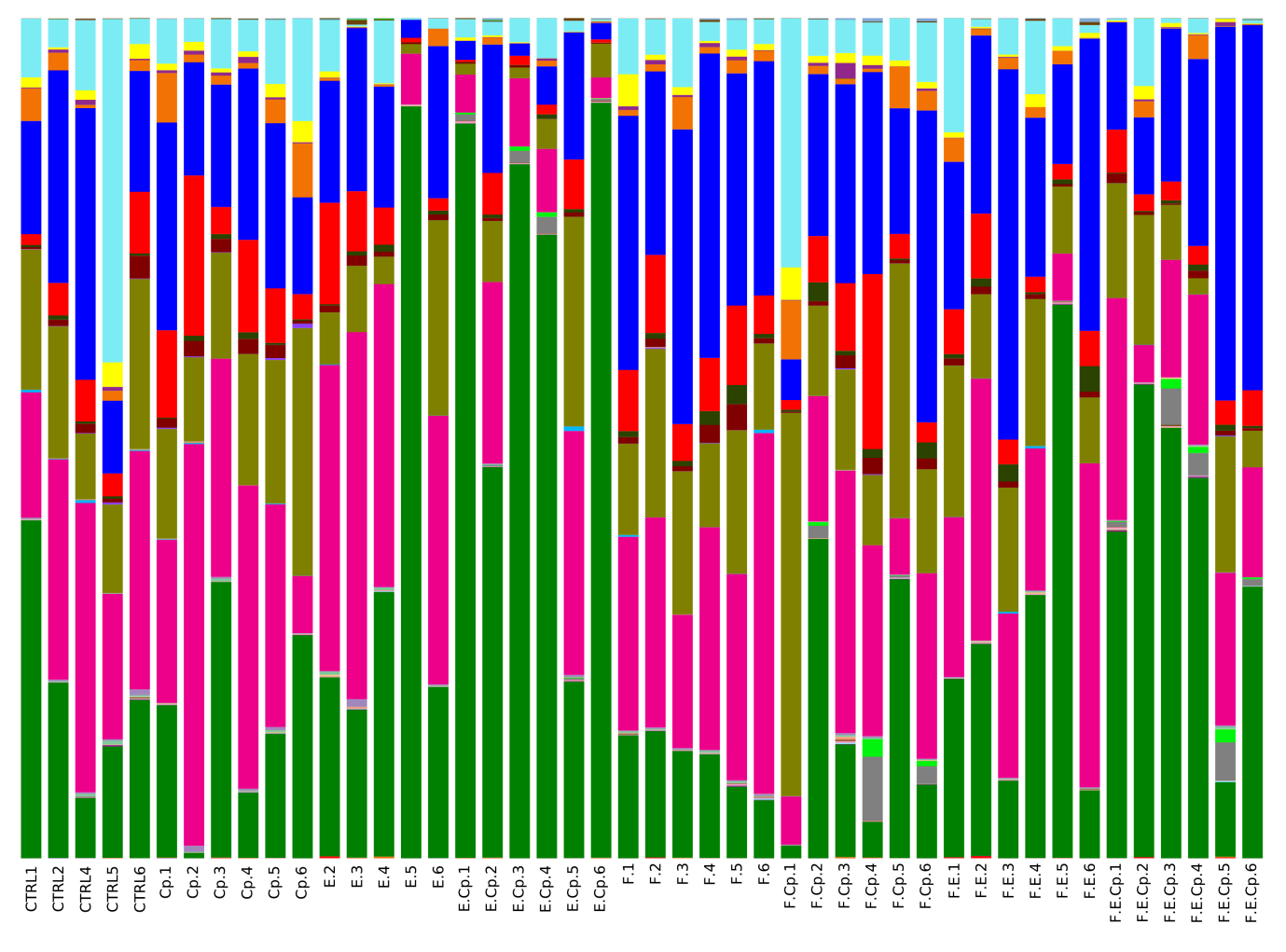


| Legend | Closest EzTaxon culturable isolate | Sequence similarity  **Note:** Only OTUs with total abundance higher than 0.001% are given in a legend.  In barchart sample names F indicates presence of fishmeal, E of *Eimeria* and Cp of *C. perfingens* challenge in a sample. |
| --- | --- | --- |
|  | *Lactobacillus johnsonii* | 99.601 |
|  | *Clostridium celerecrescens* | 96.477 |
|  | *Ethanoligenens harbinense* | 86.567 |
|  | *Clostridium papyrosolvens* | 83.333 |
|  | *Pontibacillus litoralis* | 78.895 |
|  | *Oscillibacter valericigenes* | 94.165 |
|  | *Butyricicoccus pullicaecorum* | 83.665 |
|  | *Clostridium sporosphaeroides* | 87.828 |
|  | *Acholeplasma palmae* | 79.567 |
|  | *Eubacterium desmolans* | 94.531 |
|  | *Clostridium perfringens* | 99.8 |
|  | *Eubacterium dolichum* | 85.855 |
|  | *Clostridium perfringens* | 99.572 |
|  | *Clostridium celerecrescens* | 96.477 |
|  | *Blautia hydrogenotrophica* | 97.679 |
|  | *Lactobacillus gasseri* | 86.749 |
|  | *Shigella flexneri* | 99.805 |
|  | *Roseburia hominis* | 96.252 |
|  | *Akkermansia muciniphila* | 99.798 |
|  | *Clostridium lituseburense* | 96.914 |
|  | *Lactobacillus reuteri* | 93.587 |
|  | *Mogibacterium timidum* | 88.283 |
|  | *Lactobacillus acidophilus* | 95.391 |
|  | *Flavonifractor plautii* | 99.594 |
|  | *Paenibacillus anaericanus* | 89.961 |
|  | *Enterococcus hirae* | 99.808 |
|  | *Syntrophococcus sucromutans* | 95.219 |
|  | *Weissella cibaria* | 100 |

**Figure S3:** Boxplots of OTUs identified as differential by Ade4 analysis.

Continued from main Figure 4. The boxes represent the limits of the second and third quartiles; the whiskers indicate the data within 1.5-fold of the interquartile range; the median is indicated by the horizontal lines and the dots are outliers. Note: In order to fit the legend on the x axis the names of two groups are omitted for gray column (E+FM) and for brown column (FM) in boxplots.


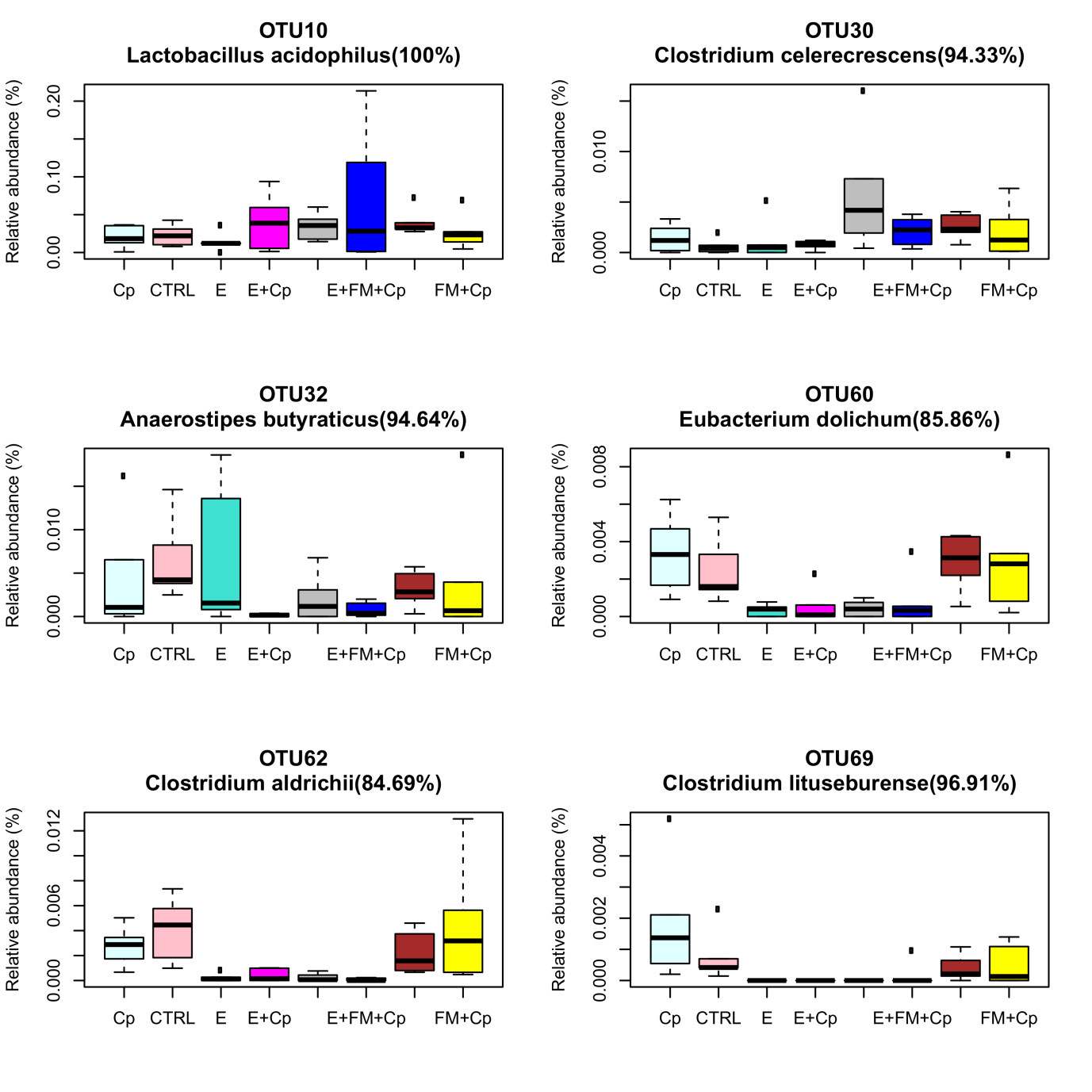


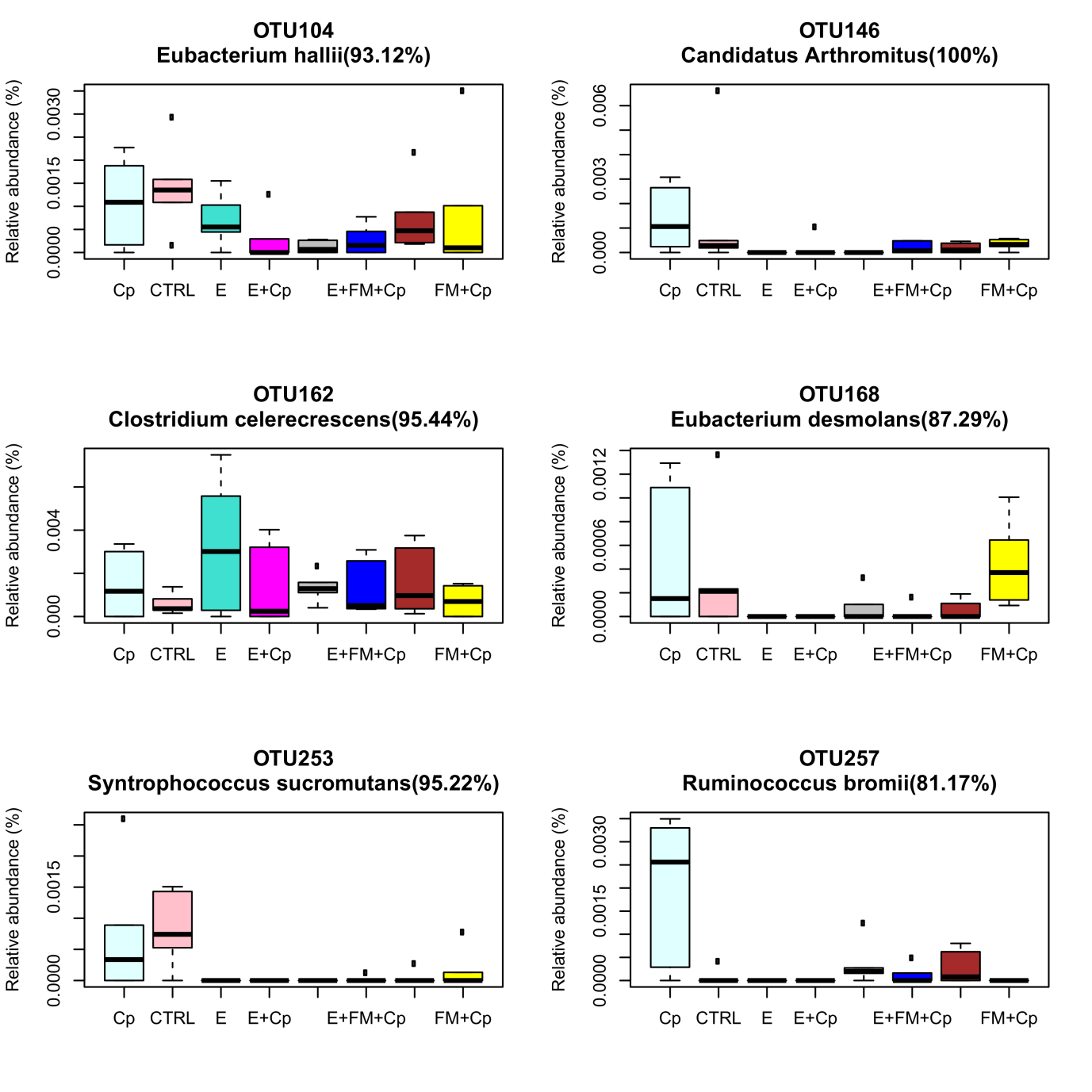

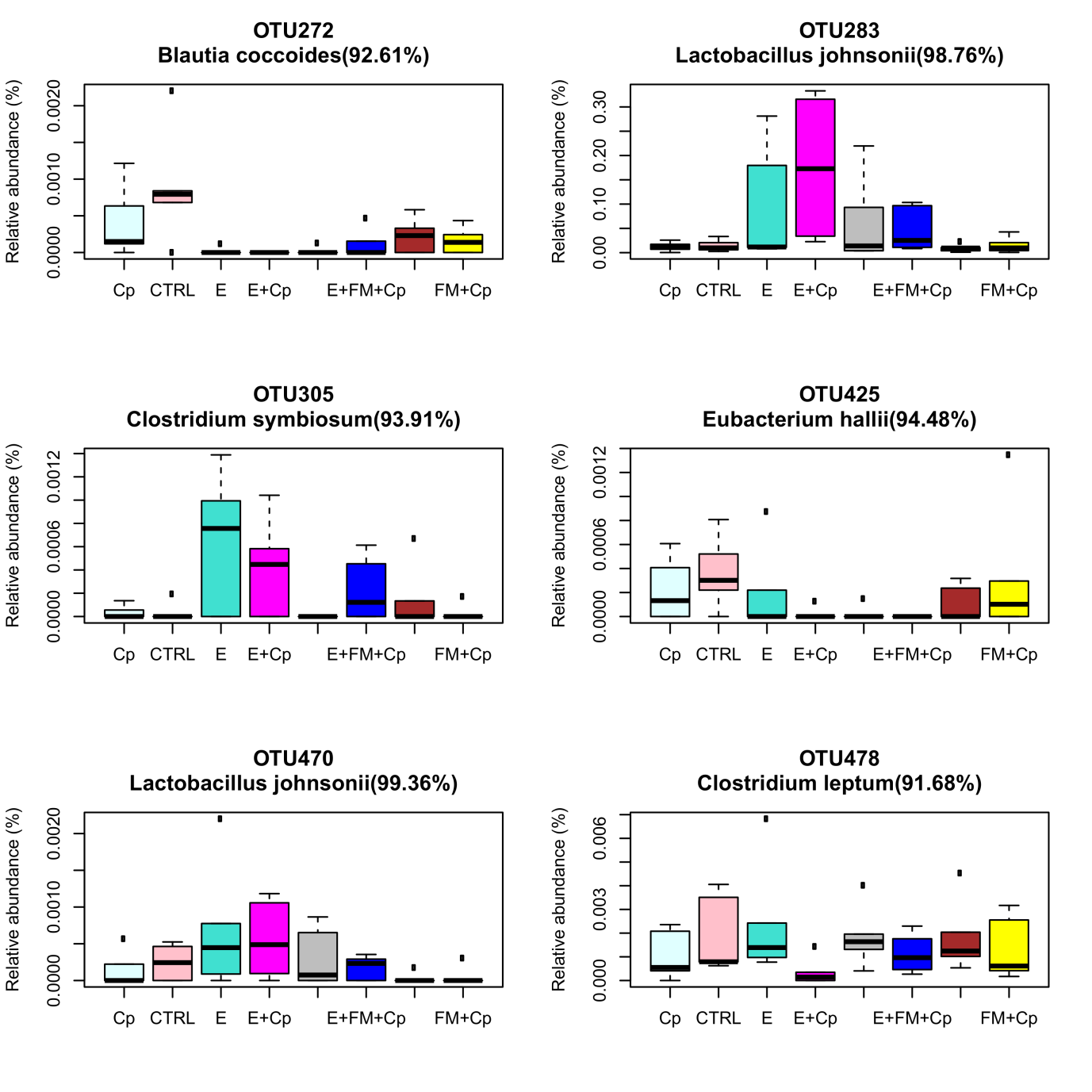


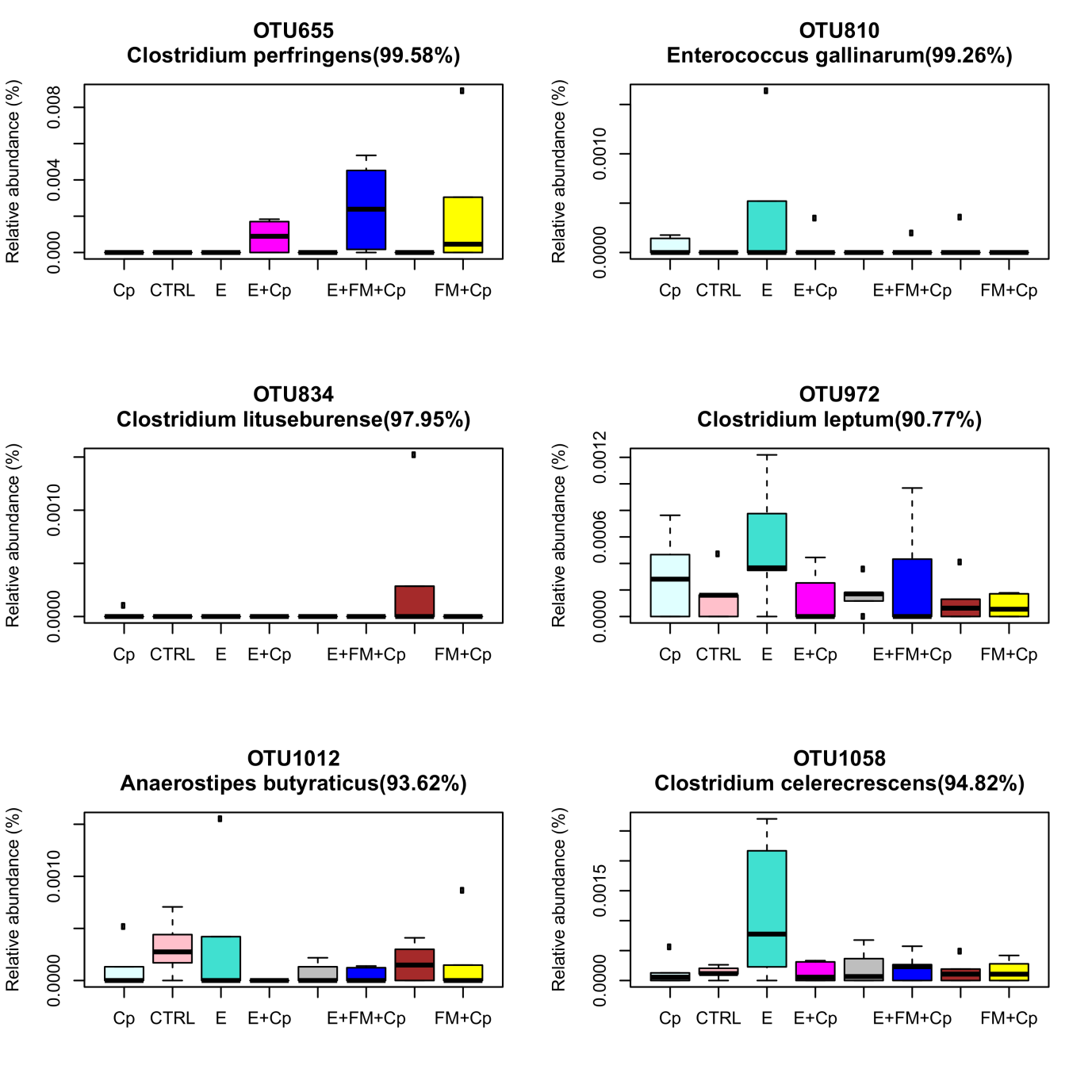

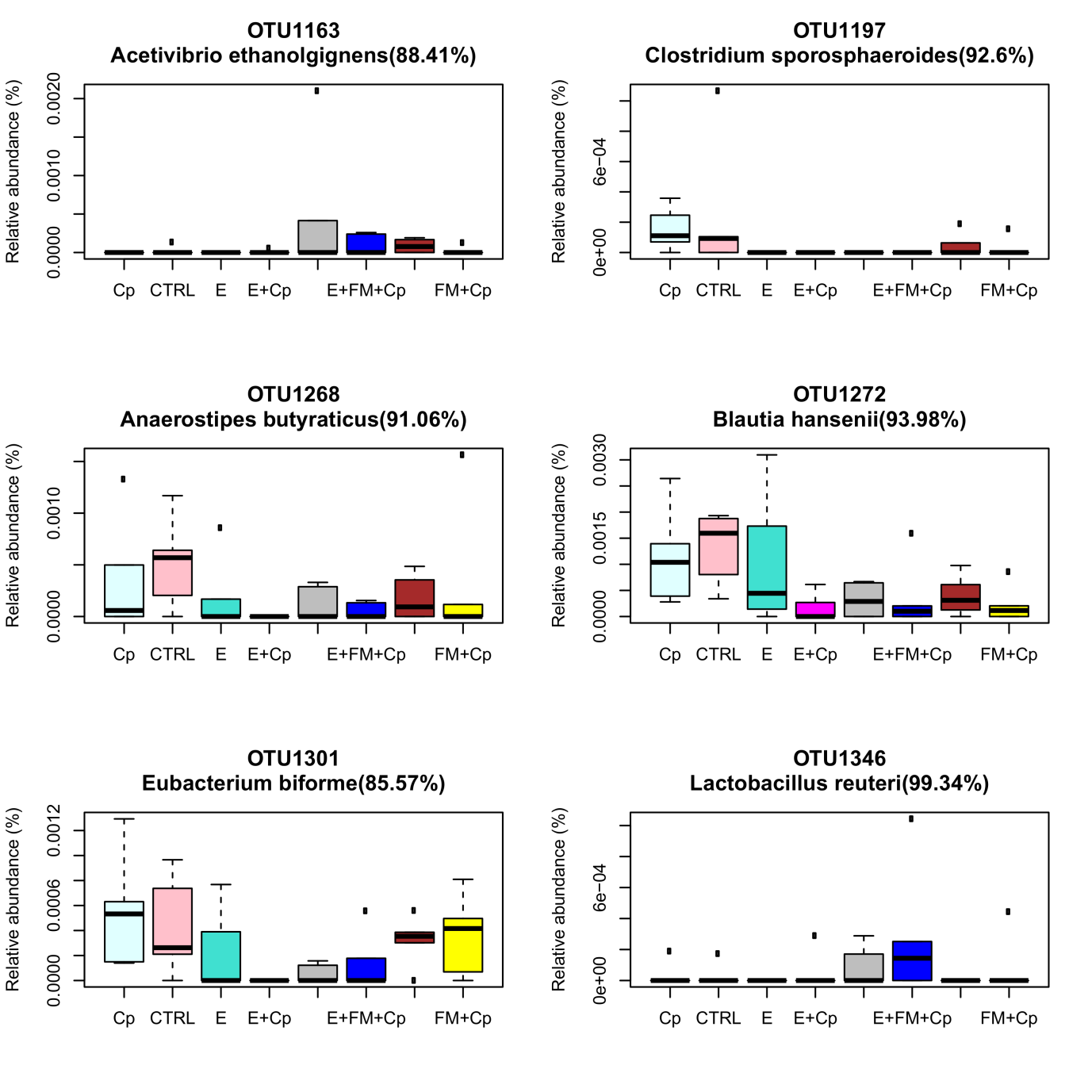

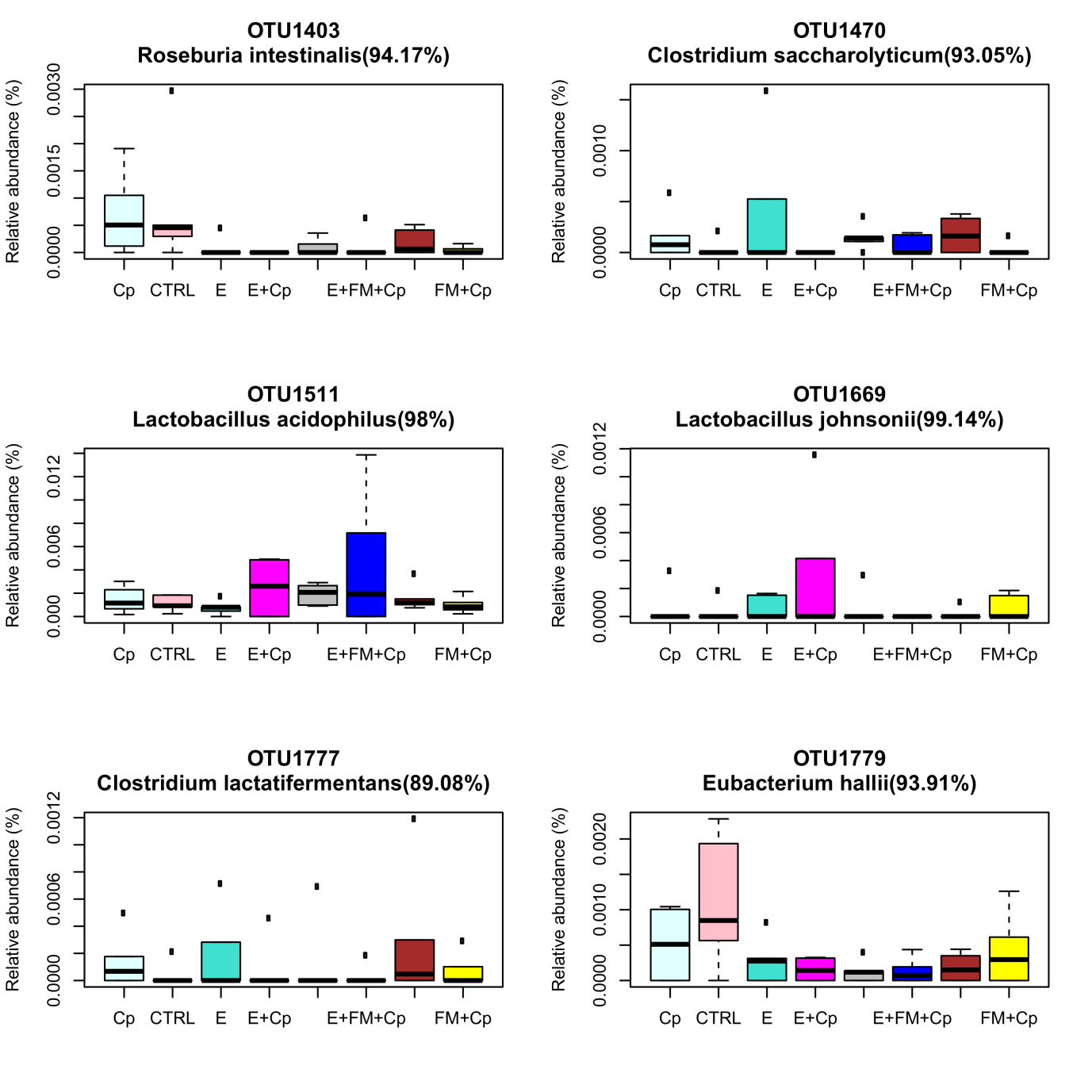

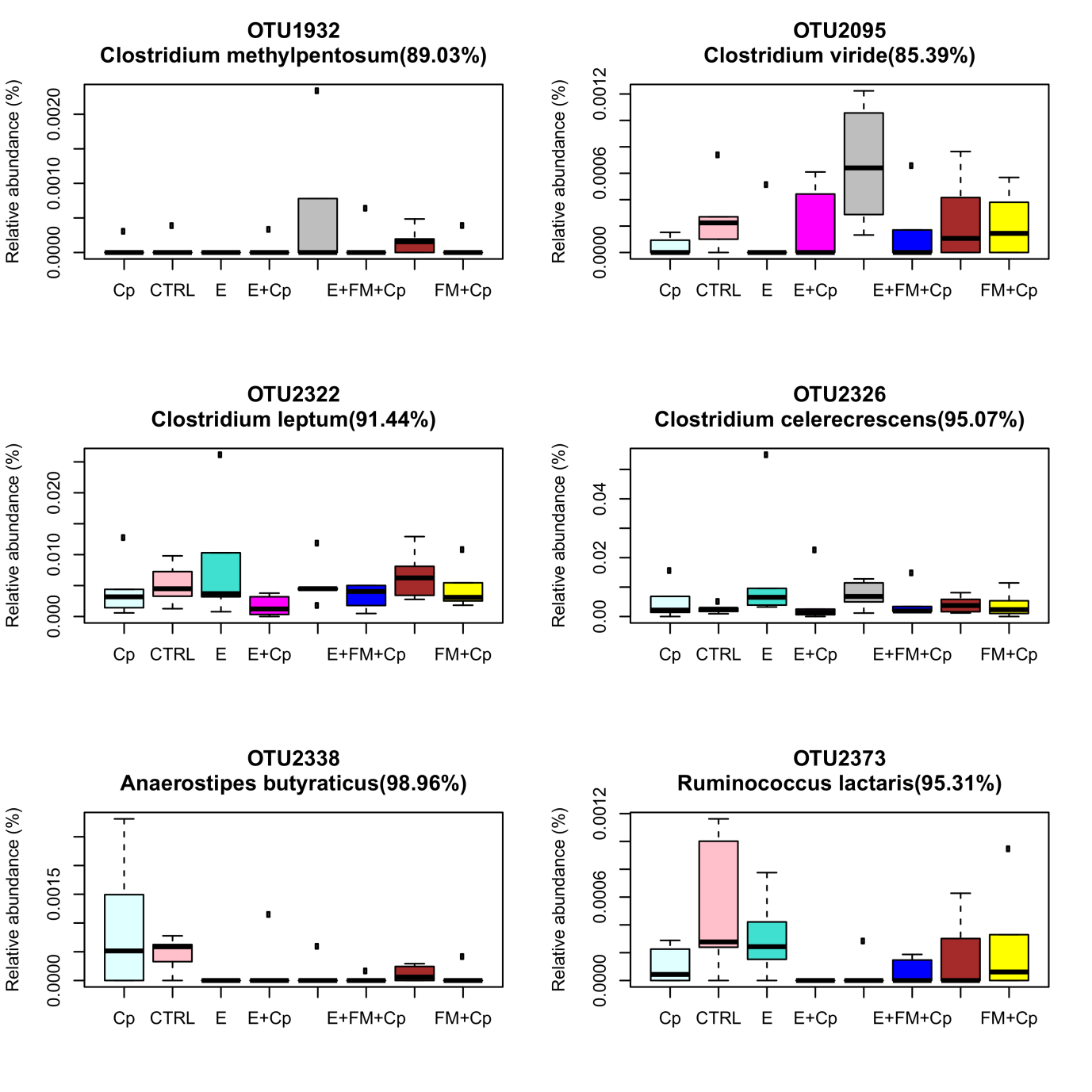

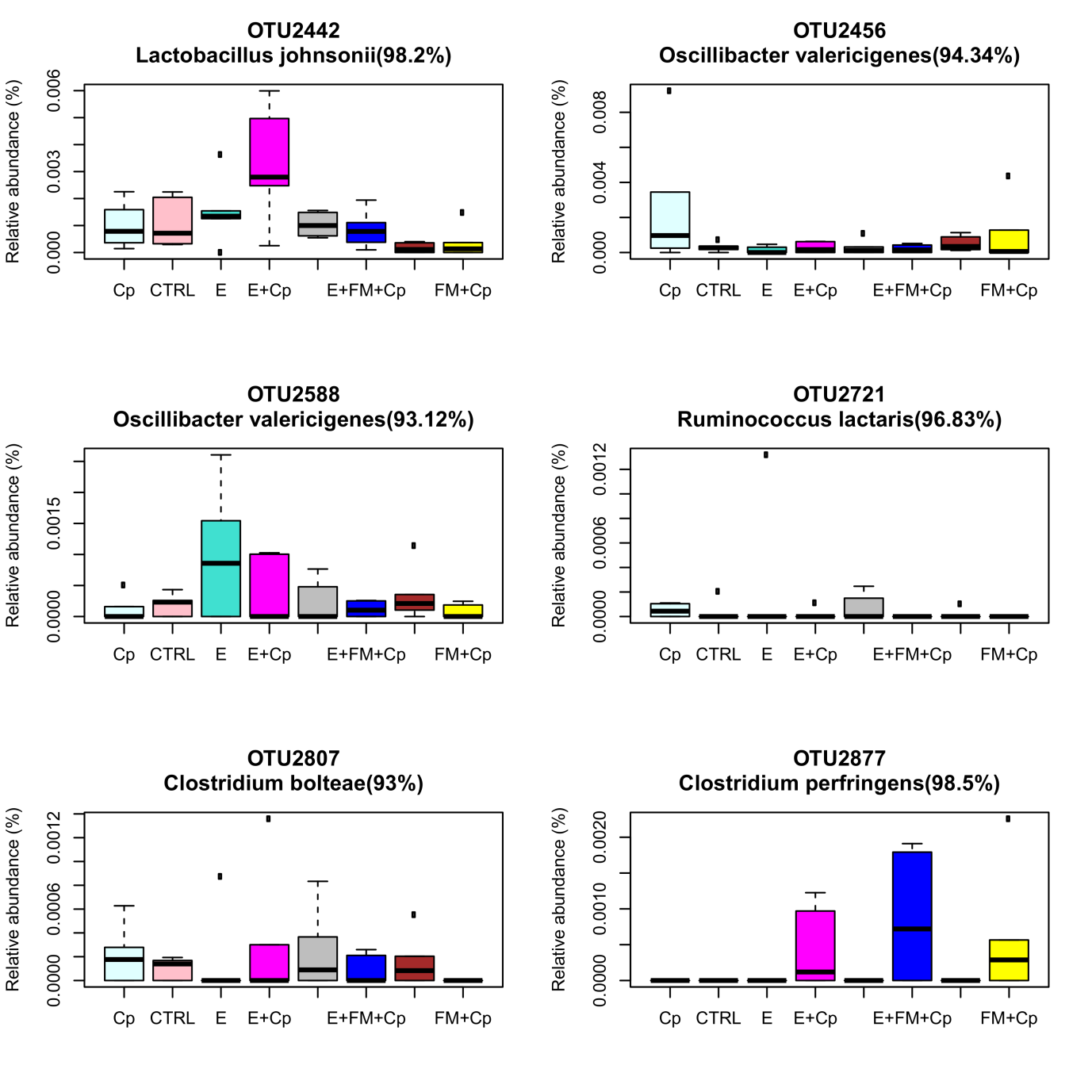

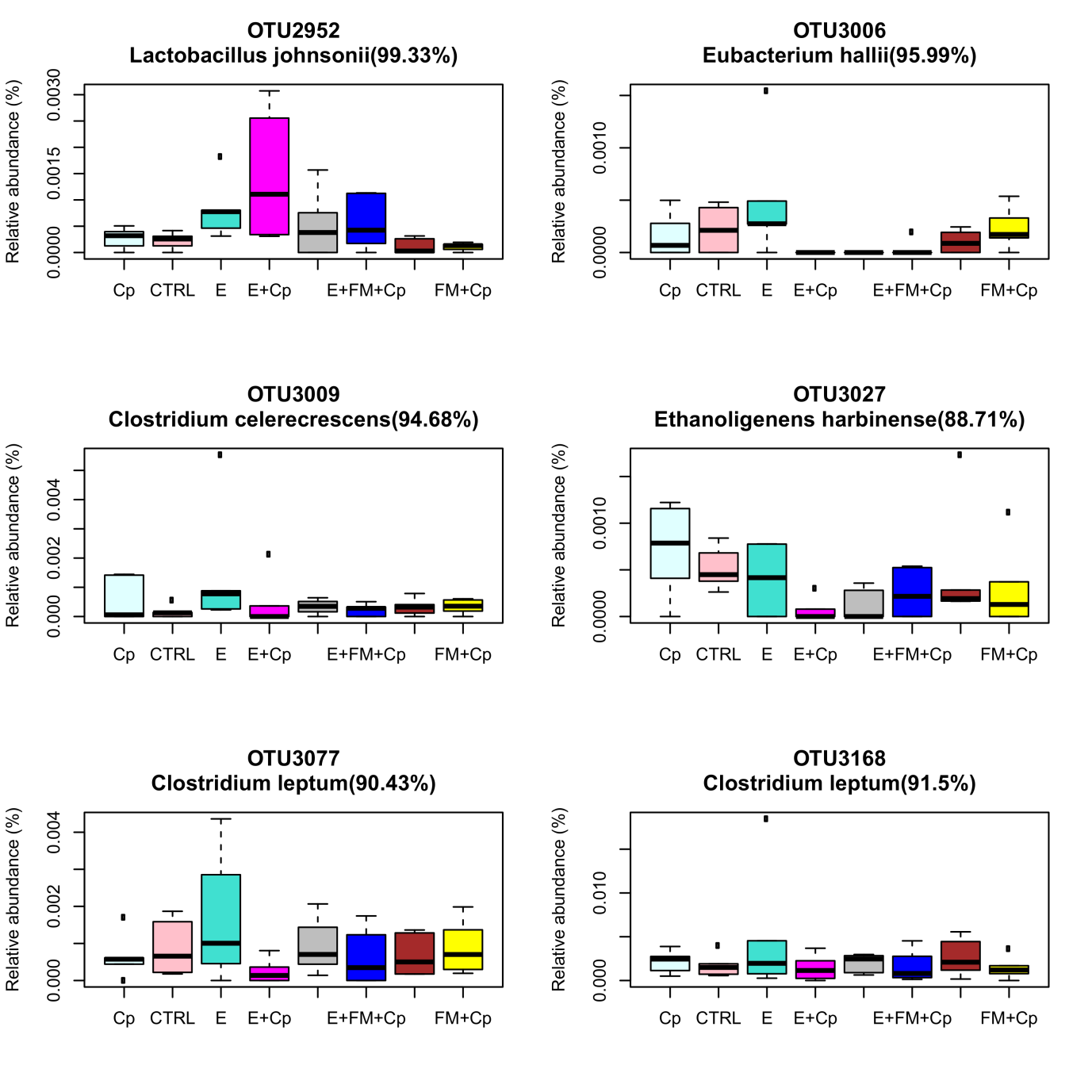


**Figure S4:** Boxplots showing SCFA, pH and cultured bacteria across the treatment groups.

The blue column in the boxplots represents E+FM+Cp group. The boxes represent the limits of the second and third quartiles; the whiskers indicate the data within 1.5-fold of the interquartile range; the median is indicated by the horizontal line and the dots are outliers.


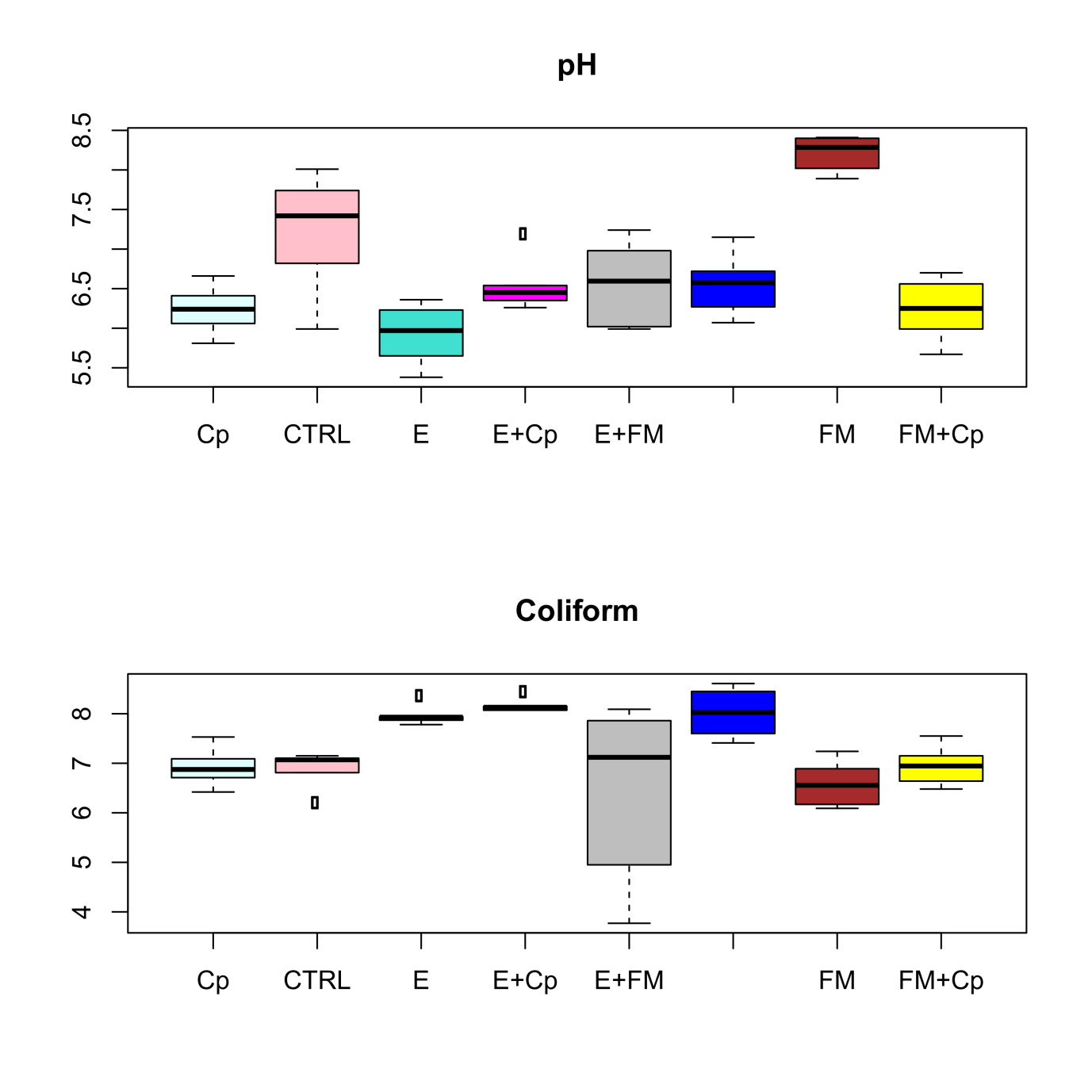


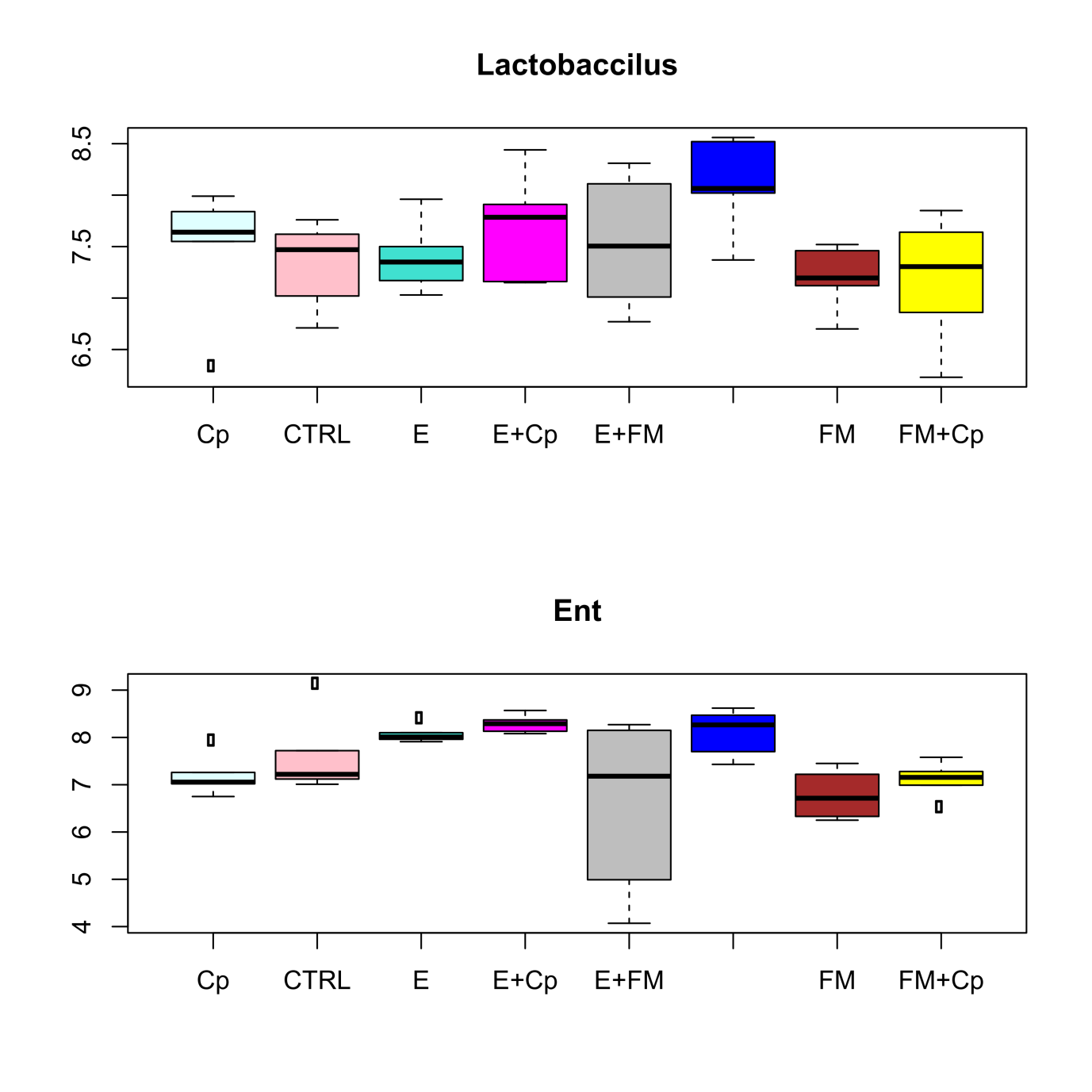


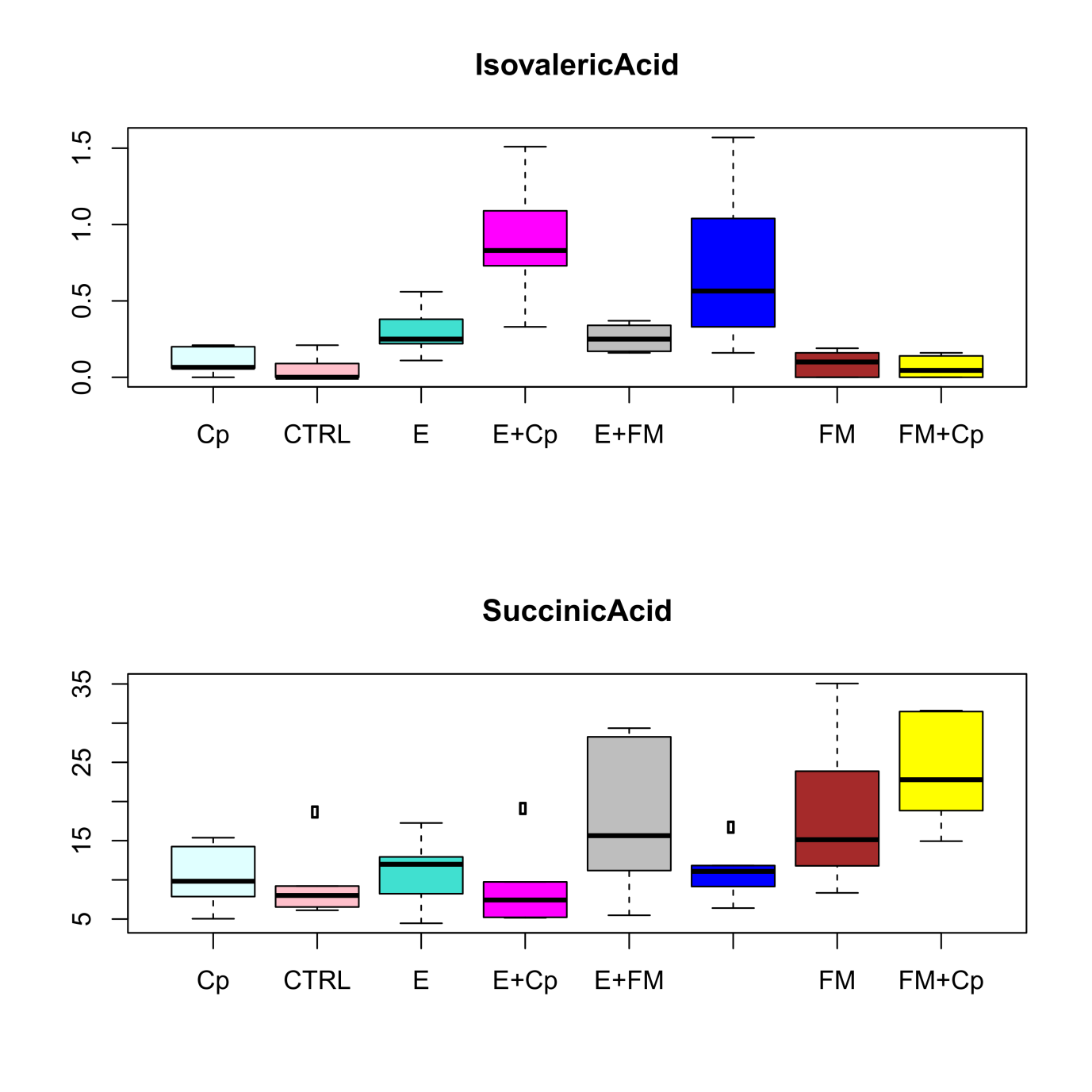


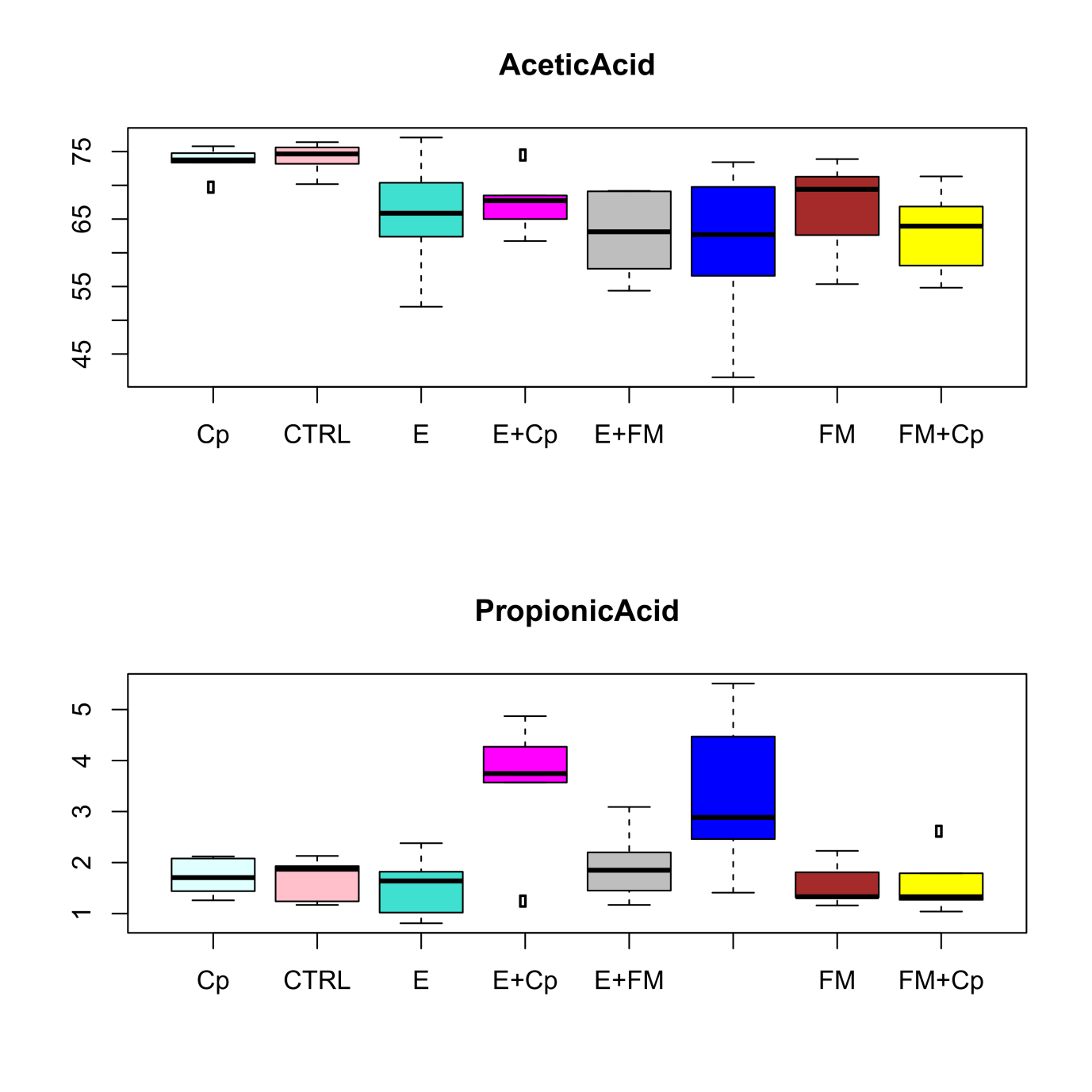


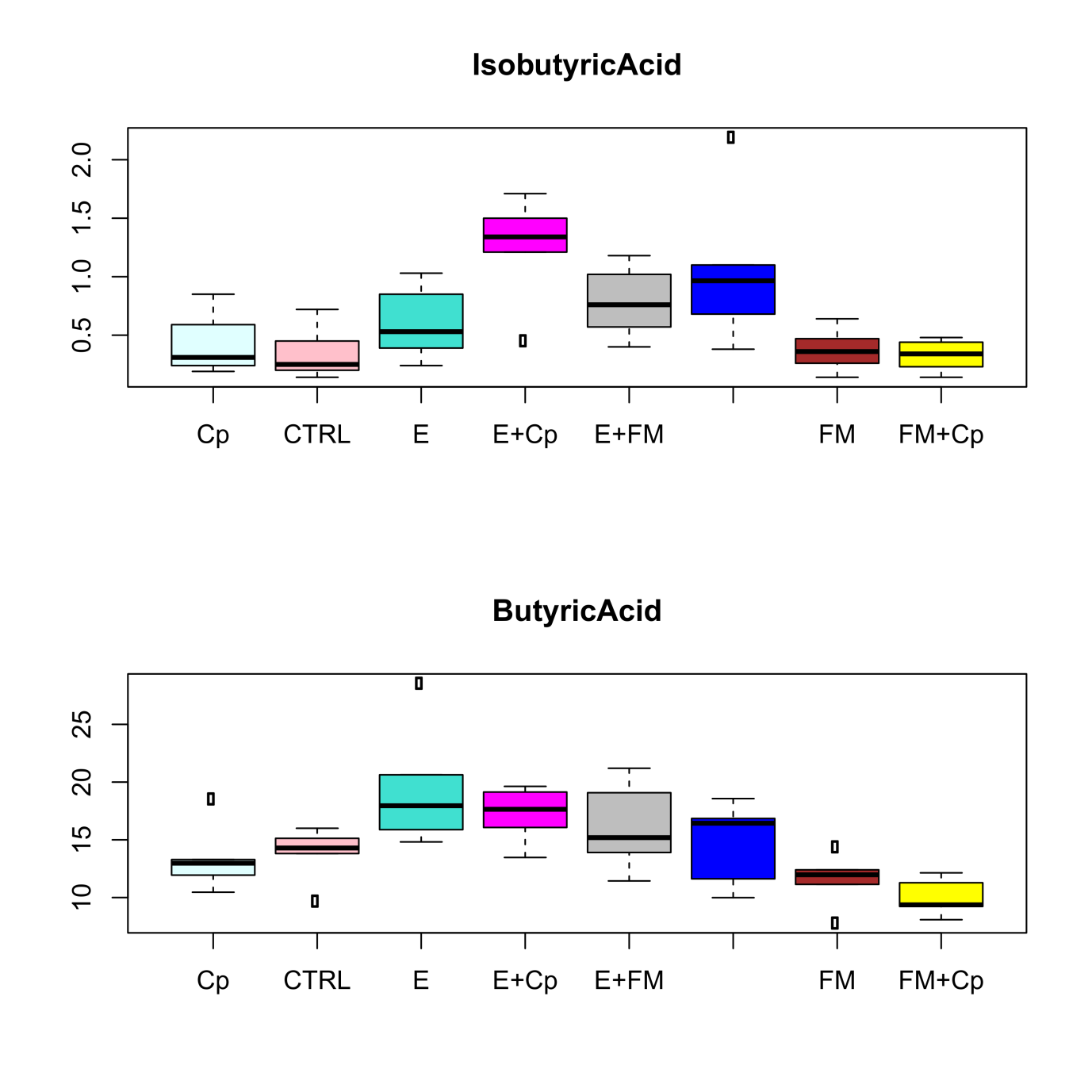

Supplement: File S1 — Contains Table S1, Comparison of alpha diversity statistics between treatment groups. The default Qiime settings were used based on a nonparametric two-sample t-test (using 100 times rarefied OTU table and 1000 Monte Carlo permutations to calculate P-values). The upper right section of the matrix, shaded light grey, indicates the p-values for the dominance metric. The lower left section of the matrix, shaded darker grey, indicates the alpha diversity comparison using the observed species metric. P values of less than 0.05 are in bold. Table S2, Detection of statistically significant differences in alpha diversity induced by the different treatments. For each comparison multiple treatment groups were combined. For example, for the Eimeria treatment comparison all birds that were treated with Eimeria, regardless of fish meal or C. perfringens treatment, were compared with all birds that did not receive Eimeria. P values of less than 0.05 are in bold. Figure S1, Alpha rarefaction graphs of groups and treatments. Alpha rarefaction measures represented as Observed Species (A and C), Dominance (B and D), Doubles (E), and Equitability (F). The analysis on individual treatment group basis is shown in panels A and B. The analysis of combined data sets (e.g. all with C. perfringens, all with Eimeria or all with both) are shown in panels C–F and demonstrate the strong influence by the combination of Eimeria and C. perfringens. Figure S2, Barchart of OTU abundances at a species level. Figure S3, Boxplots of OTUs identified as differential by Ade4 analysis. Continued from main Figure 4. The boxes represent the limits of the second and third quartiles; the whiskers indicate the data within 1.5-fold of the interquartile range; the median is indicated by the horizontal line and the dots are outliers. Note: In order to fit the legend on the x axis the names of two groups are omitted for gray column (E+FM) and for brown column (FM) in boxplots. Figure S4, Boxplots showing SCFA, pH an [file pone.0104739.s001.docx]
